# Supplementary material for: Altered microRNA Transcriptome in Cultured Human Liver Cells upon Infection with Ebola Virus
Source: Int J Mol Sci. 2021 Apr 6;22(7):3792. doi: 10.3390/ijms22073792 (PMC8038836; doi:10.3390/ijms22073792)
Supplement: Supplementary file 1 [file ijms-22-03792-s001.zip › Supplementary_File/C_ GO_Analysis_Results/16-30nt_go_Mayinga-24h-Huh7_vs_Control-24h-Huh7_down.mature_mirna_targets/BP_result(Human).html]

| GO.ID | Term | Ontology | Count | Pop.Hits | List.Total | Pop.Total | Fold.Enrichment | Pvalue | FDR | Enrichment.Score | Gene.Ratio | GENES |
| --- | --- | --- | --- | --- | --- | --- | --- | --- | --- | --- | --- | --- |
| GO:0007411 | axon guidance | Biological process | 12 | 248 | 169 | 17653 | 5.05430425653751 | 4.91167166608656e-06 | 0.0180299926215688 | 5.30877067248023 | 0.0710059171597633 | UNC5A//SEMA4B//SEMA4G//ZSWIM6//EFNA3//GPC1//ARHGAP35//NCAM1//SPTBN5//PRKCQ//BDNF//CNTN2// |
| GO:0097485 | neuron projection guidance | Biological process | 12 | 250 | 169 | 17653 | 5.01386982248521 | 5.33195109317428e-06 | 0.0180299926215688 | 5.27311384276567 | 0.0710059171597633 | EFNA3//GPC1//ARHGAP35//NCAM1//SPTBN5//PRKCQ//BDNF//CNTN2//UNC5A//SEMA4B//SEMA4G//ZSWIM6// |
| GO:0007409 | axonogenesis | Biological process | 15 | 456 | 169 | 17653 | 3.4360401744005 | 3.37354109642123e-05 | 0.0716095635405452 | 4.47191399490834 | 0.0887573964497041 | EFNA3//GPC1//ARHGAP35//NCAM1//SPTBN5//PRKCQ//BDNF//CNTN2//UNC5A//SRF//SEMA4B//SEMA4G//NPTX1//ZSWIM6//APBB1// |
| GO:0120036 | plasma membrane bounded cell projection organization | Biological process | 30 | 1444 | 169 | 17653 | 2.17013063646347 | 4.2353726772465e-05 | 0.0716095635405452 | 4.3731083693827 | 0.177514792899408 | APBB1//EFNA3//GPC1//ARHGAP35//NCAM1//SPTBN5//PRKCQ//BDNF//CNTN2//UNC5A//MCIDAS//FAM161A//DYNLL1//TWF1//BRK1//SFRP1//ARFIP2//ARHGEF6//UBE4B//GNAO1//SRF//BTG2//VAPA//CAMK2A//SEMA4B//SEMA4G//NPTX1//ZSWIM6//ABHD17B//GRIN2B// |
| GO:0030030 | cell projection organization | Biological process | 30 | 1477 | 169 | 17653 | 2.12164430538474 | 6.41861502743197e-05 | 0.0861563038481793 | 4.19255867143766 | 0.177514792899408 | APBB1//EFNA3//GPC1//ARHGAP35//NCAM1//SPTBN5//PRKCQ//BDNF//CNTN2//UNC5A//MCIDAS//FAM161A//DYNLL1//TWF1//BRK1//SFRP1//ARFIP2//ARHGEF6//UBE4B//GNAO1//SRF//BTG2//VAPA//CAMK2A//SEMA4B//SEMA4G//NPTX1//ZSWIM6//ABHD17B//GRIN2B// |
| GO:0031175 | neuron projection development | Biological process | 22 | 936 | 169 | 17653 | 2.45515349213574 | 8.87344147730607e-05 | 0.0861563038481793 | 4.05190791063836 | 0.130177514792899 | APBB1//EFNA3//GPC1//ARHGAP35//NCAM1//SPTBN5//PRKCQ//BDNF//CNTN2//UNC5A//SFRP1//TWF1//SRF//CAMK2A//SEMA4B//SEMA4G//NPTX1//ZSWIM6//UBE4B//GNAO1//BTG2//VAPA// |
| GO:0061564 | axon development | Biological process | 15 | 497 | 169 | 17653 | 3.15258414391676 | 8.91755325945964e-05 | 0.0861563038481793 | 4.04975428818862 | 0.0887573964497041 | APBB1//EFNA3//GPC1//ARHGAP35//NCAM1//SPTBN5//PRKCQ//BDNF//CNTN2//UNC5A//SRF//SEMA4B//SEMA4G//NPTX1//ZSWIM6// |
| GO:0048667 | cell morphogenesis involved in neuron differentiation | Biological process | 16 | 567 | 169 | 17653 | 2.94760130657567 | 0.000113154749249669 | 0.0956581961469389 | 3.9463272134314 | 0.0946745562130177 | APBB1//EFNA3//GPC1//ARHGAP35//NCAM1//SPTBN5//PRKCQ//BDNF//CNTN2//UNC5A//SRF//CAMK2A//SEMA4B//SEMA4G//NPTX1//ZSWIM6// |
| GO:0000904 | cell morphogenesis involved in differentiation | Biological process | 18 | 701 | 169 | 17653 | 2.68217001916113 | 0.000137786845719316 | 0.103539159733304 | 3.86079224182029 | 0.106508875739645 | APBB1//EFNA3//GPC1//ARHGAP35//NCAM1//SPTBN5//PRKCQ//BDNF//CNTN2//UNC5A//EP300//SRF//FERMT3//CAMK2A//SEMA4B//SEMA4G//NPTX1//ZSWIM6// |
| GO:0048812 | neuron projection morphogenesis | Biological process | 16 | 628 | 169 | 17653 | 2.66128971469491 | 0.000357899374749081 | 0.242047347142803 | 3.44623906032266 | 0.0946745562130177 | APBB1//EFNA3//GPC1//ARHGAP35//NCAM1//SPTBN5//PRKCQ//BDNF//CNTN2//UNC5A//SRF//CAMK2A//SEMA4B//SEMA4G//NPTX1//ZSWIM6// |
| GO:0043543 | protein acylation | Biological process | 9 | 233 | 169 | 17653 | 4.0347664880514 | 0.000418196210333235 | 0.244801926661581 | 3.37861990704877 | 0.0532544378698225 | EP300//FOXO1//HAT1//ZDHHC22//ABHD17B//FOXP3//APBB1//OGT//FAM161A// |
| GO:0120039 | plasma membrane bounded cell projection morphogenesis | Biological process | 16 | 642 | 169 | 17653 | 2.6032553595458 | 0.000455554074350833 | 0.244801926661581 | 3.34146006479782 | 0.0946745562130177 | APBB1//EFNA3//GPC1//ARHGAP35//NCAM1//SPTBN5//PRKCQ//BDNF//CNTN2//UNC5A//SRF//CAMK2A//SEMA4B//SEMA4G//NPTX1//ZSWIM6// |
| GO:0048858 | cell projection morphogenesis | Biological process | 16 | 644 | 169 | 17653 | 2.59517071557205 | 0.000471225447163238 | 0.244801926661581 | 3.32677126477408 | 0.0946745562130177 | APBB1//EFNA3//GPC1//ARHGAP35//NCAM1//SPTBN5//PRKCQ//BDNF//CNTN2//UNC5A//SRF//CAMK2A//SEMA4B//SEMA4G//NPTX1//ZSWIM6// |
| GO:0048666 | neuron development | Biological process | 22 | 1060 | 169 | 17653 | 2.16794685720665 | 0.000506761344560423 | 0.244801926661581 | 3.29519652023763 | 0.130177514792899 | APBB1//EFNA3//GPC1//ARHGAP35//NCAM1//SPTBN5//PRKCQ//BDNF//CNTN2//UNC5A//SFRP1//TWF1//BTG2//UBE4B//GNAO1//SRF//VAPA//CAMK2A//SEMA4B//SEMA4G//NPTX1//ZSWIM6// |
| GO:0000902 | cell morphogenesis | Biological process | 21 | 997 | 169 | 17653 | 2.20016855299627 | 0.000565895785914196 | 0.254811359394173 | 3.24726354013611 | 0.124260355029586 | APBB1//EFNA3//GPC1//ARHGAP35//NCAM1//SPTBN5//PRKCQ//BDNF//CNTN2//UNC5A//RHOF//ENAM//EP300//SRF//FERMT3//CAMK2A//SEMA4B//SEMA4G//NPTX1//ZSWIM6//CDH7// |
| GO:0032990 | cell part morphogenesis | Biological process | 16 | 663 | 169 | 17653 | 2.52079930743349 | 0.000644780173118606 | 0.254811359394173 | 3.19058832547959 | 0.0946745562130177 | APBB1//EFNA3//GPC1//ARHGAP35//NCAM1//SPTBN5//PRKCQ//BDNF//CNTN2//UNC5A//SRF//CAMK2A//SEMA4B//SEMA4G//NPTX1//ZSWIM6// |
| GO:0032535 | regulation of cellular component size | Biological process | 11 | 358 | 169 | 17653 | 3.20953026346236 | 0.000682259501289596 | 0.254811359394173 | 3.16605040753947 | 0.0650887573964497 | TWF1//ARHGAP35//BRK1//CNTN2//ARFIP2//SRF//BDNF//SEMA4B//SEMA4G//SPTBN5//RAB3B// |
| GO:0048585 | negative regulation of response to stimulus | Biological process | 29 | 1615 | 169 | 17653 | 1.87567369520215 | 0.000687980521160834 | 0.254811359394173 | 3.16242385779955 | 0.171597633136095 | FOXP3//MIF//MGRN1//YWHAB//SMPD1//TRABD2B//SFRP1//FAM89B//TOB1//CTDSPL2//SH3BP4//PRKAA1//FOXO1//ARHGAP35//GPATCH3//GPC1//IL4R//PRKCQ//SEMA4B//SEMA4G//CGNL1//HEYL//GPD1L//PSMB8//DDAH1//PTPN18//CDKN2D//OSR1//LYPD6// |
| GO:0007165 | signal transduction | Biological process | 80 | 6210 | 169 | 17653 | 1.34564407474106 | 0.000715868080510024 | 0.254811359394173 | 3.14516700169365 | 0.473372781065089 | GRIN2B//NCAM1//SPTBN5//PSMB8//YWHAB//CAMK2A//PRKAA1//EP300//TRABD2B//NDP//GNAO1//PTPRT//BDNF//CARHSP1//PRKCQ//SH3BP5//BTG2//MIF//CLEC1A//CHRM1//ABCA1//GNAZ//GPR153//SFRP1//ARHGEF6//GPR3//HEYL//EVC//ADAM11//FERMT3//OSR1//DDAH1//RALBP1//ARFIP2//RHOF//FOXO1//FGFRL1//POLR2E//UBE4B//TOB1//CSPG5//MGRN1//BRK1//IL4R//FOXP3//SMPD1//NPNT//FAM89B//CTDSPL2//CREB3L3//SH3BP4//RABL3//RAB3B//KMT2D//OGT//ARHGAP35//P2RX2//GOSR2//UNC5A//PTPN18//PAX8//GPATCH3//GPC1//VAPA//CRIM1//EFNA3//CGNL1//CNTN2//SEMA4B//SEMA4G//ZNF385B//GPD1L//BSN//LYPD6//DYNLL1//CDKN2D//CNIH2//AKAP10//KCNIP3//APBB1// |
| GO:0023052 | signaling | Biological process | 85 | 6724 | 169 | 17653 | 1.320453273446 | 0.000782961598057463 | 0.264758464383131 | 3.10625953827504 | 0.502958579881657 | GRIN2B//NCAM1//SPTBN5//PSMB8//YWHAB//CAMK2A//PRKAA1//EP300//EFNA3//NDP//BDNF//TRABD2B//GNAO1//RALBP1//PTPRT//AKAP10//CHRM1//ARHGAP35//KCNIP3//APBB1//IL4R//FGFRL1//SMPD1//OGT//VAPA//SH3BP5//CARHSP1//PRKCQ//BTG2//MIF//CLEC1A//ABCA1//GNAZ//GPR153//SFRP1//ARHGEF6//GPR3//HEYL//EVC//ADAM11//FERMT3//OSR1//DDAH1//ARFIP2//RHOF//CPLX1//NPTX1//BSN//P2RX2//FOXO1//POLR2E//UBE4B//TOB1//CSPG5//MGRN1//BRK1//FOXP3//NPNT//FAM89B//CTDSPL2//CREB3L3//SH3BP4//RABL3//RAB3B//KMT2D//CNIH2//DYNLL1//GOSR2//UNC5A//PTPN18//PAX8//GPATCH3//GPC1//CRIM1//RIMS4//CNTN2//CGNL1//SRF//SEMA4B//SEMA4G//ZNF385B//SCN3B//GPD1L//LYPD6//CDKN2D// |
| GO:0060341 | regulation of cellular localization | Biological process | 18 | 815 | 169 | 17653 | 2.30699531709442 | 0.000833690598805591 | 0.267188067139483 | 3.07899509583999 | 0.106508875739645 | P2RX2//DYNLL1//IL4R//NUP214//CTDSPL2//CPLX1//CAMK2A//RAB3B//CHRM1//YWHAB//CNIH2//ABHD17B//NUCB1//TP53INP2//PRKAA1//BDNF//GPD1L//RIMS4// |
| GO:0007154 | cell communication | Biological process | 85 | 6744 | 169 | 17653 | 1.31653733847137 | 0.000870442364096634 | 0.267188067139483 | 3.06025998018534 | 0.502958579881657 | GRIN2B//NCAM1//SPTBN5//PSMB8//YWHAB//CAMK2A//PRKAA1//EP300//EFNA3//NDP//BDNF//TRABD2B//GNAO1//RALBP1//PTPRT//AKAP10//CHRM1//ARHGAP35//KCNIP3//APBB1//IL4R//FGFRL1//SMPD1//OGT//VAPA//SH3BP5//CARHSP1//PRKCQ//BTG2//MIF//CLEC1A//ABCA1//GNAZ//GPR153//SFRP1//ARHGEF6//GPR3//HEYL//EVC//ADAM11//FERMT3//OSR1//DDAH1//ARFIP2//RHOF//CPLX1//NPTX1//BSN//P2RX2//FOXO1//POLR2E//UBE4B//TOB1//CSPG5//MGRN1//BRK1//FOXP3//NPNT//FAM89B//CTDSPL2//CREB3L3//SH3BP4//RABL3//RAB3B//KMT2D//CNIH2//DYNLL1//GOSR2//UNC5A//PTPN18//PAX8//GPATCH3//GPC1//SRF//CRIM1//RIMS4//CNTN2//CGNL1//SEMA4B//SEMA4G//ZNF385B//SCN3B//GPD1L//LYPD6//CDKN2D// |
| GO:0007399 | nervous system development | Biological process | 37 | 2296 | 169 | 17653 | 1.68330051749376 | 0.000908668570783395 | 0.267188067139483 | 3.04159449315514 | 0.218934911242604 | SRF//ARHGAP35//APBB1//EFNA3//GPC1//NCAM1//SPTBN5//PRKCQ//BDNF//CNTN2//UNC5A//BSN//NPTX1//PAX8//GRIN2B//SFRP1//TWF1//HDAC11//BTG2//DYNLL1//GNAO1//UBE4B//VAPA//HEYL//CAMK2A//SEMA4B//SEMA4G//ZSWIM6//CSPG5//CHRM1//EP300//KIF2A//NDP//CRIM1//SCN3B//PURA//SMPD1// |
| GO:0071840 | cellular component organization or biogenesis | Biological process | 82 | 6486 | 169 | 17653 | 1.32059219037089 | 0.00108472758877537 | 0.305667195120326 | 2.96467931393139 | 0.485207100591716 | UBXN2B//TP53INP2//THUMPD1//KIF2A//CNTN2//SHQ1//COPS7B//CDH7//CRIM1//PRKCQ//RHOF//TWF1//MICAL2//ARFIP2//SPTBN5//PFDN1//NDP//PURA//HAT1//HDAC11//ASF1B//FOXP3//BAHD1//KMT2D//RPP14//TOMM40//ABCA1//BRK1//CGNL1//DYNLL1//NUP214//APBB1//EFNA3//GPC1//ARHGAP35//NCAM1//BDNF//UNC5A//BSN//P2RX2//VAPA//MCIDAS//FAM161A//ATXN7L3//SFRP1//ISCU//EP300//ARID4B//ENAM//ARHGEF6//NPNT//SRF//CDKN2D//SH3BP4//UBE4B//GNAO1//BTG2//TRABD2B//FERMT3//FAF2//PRKAA1//VPS37D//GPR3//KCTD18//FGFRL1//OGT//CNIH2//GOSR2//CAMK2A//SEMA4B//SEMA4G//DPYS//GRIN2B//YWHAB//NPTX1//ANO3//MIF//RAB3B//IL4R//ZSWIM6//ABHD17B//NUDT16// |
| GO:0050770 | regulation of axonogenesis | Biological process | 7 | 167 | 169 | 17653 | 4.37837933600255 | 0.00114328522435489 | 0.309281518892485 | 2.94184540922802 | 0.0414201183431953 | CNTN2//SRF//BDNF//SEMA4B//SEMA4G//ZSWIM6//ARHGAP35// |
| GO:0033036 | macromolecule localization | Biological process | 44 | 2955 | 169 | 17653 | 1.55534596862203 | 0.00140026147713006 | 0.351152874557435 | 2.853790858985 | 0.260355029585799 | SPRN//NUP214//TWF1//AKAP10//SRSF3//YWHAB//ZDHHC22//GOSR2//TOMM40//ABCA1//PCTP//ANO3//ARFIP2//RABL3//KCNIP3//RAB3B//MLPH//VPS37D//VPS53//CPLX1//FAF2//CYP4F2//BSN//DYNLL1//NPNT//SFRP1//CTDSPL2//FOXP3//APBB1//MIF//SH3BP4//TULP4//VAPA//CNTN2//SCN3B//IL4R//CHRM1//ABHD17B//NUCB1//TP53INP2//PRKAA1//SHQ1//BDNF//GPD1L// |
| GO:0060538 | skeletal muscle organ development | Biological process | 7 | 173 | 169 | 17653 | 4.2265280295516 | 0.00140191152048658 | 0.351152874557435 | 2.85327939534013 | 0.0414201183431953 | EP300//HEYL//BTG2//PRKAA1//P2RX2//FGFRL1//GPC1// |
| GO:0008104 | protein localization | Biological process | 40 | 2628 | 169 | 17653 | 1.58988769104681 | 0.00162987571271697 | 0.384543842130487 | 2.78784551175505 | 0.236686390532544 | SPRN//NUP214//TWF1//SRSF3//YWHAB//ZDHHC22//GOSR2//TOMM40//ARFIP2//RABL3//KCNIP3//RAB3B//MLPH//VPS37D//VPS53//CPLX1//FAF2//BSN//DYNLL1//NPNT//SFRP1//CTDSPL2//ABCA1//FOXP3//APBB1//MIF//SH3BP4//TULP4//VAPA//CNTN2//SCN3B//IL4R//CHRM1//ABHD17B//NUCB1//TP53INP2//PRKAA1//BDNF//GPD1L//AKAP10// |
| GO:0016043 | cellular component organization | Biological process | 79 | 6269 | 169 | 17653 | 1.31631744821187 | 0.00164893855120274 | 0.384543842130487 | 2.78279552832808 | 0.467455621301775 | UBXN2B//TP53INP2//KIF2A//CNTN2//SHQ1//COPS7B//CDH7//CRIM1//PRKCQ//RHOF//TWF1//MICAL2//ARFIP2//SPTBN5//PFDN1//NDP//PURA//HAT1//HDAC11//ASF1B//FOXP3//BAHD1//KMT2D//TOMM40//ABCA1//BRK1//CGNL1//DYNLL1//NUP214//APBB1//EFNA3//GPC1//ARHGAP35//NCAM1//BDNF//UNC5A//BSN//P2RX2//VAPA//MCIDAS//FAM161A//ATXN7L3//SFRP1//ISCU//EP300//ARID4B//ENAM//ARHGEF6//NPNT//SRF//CDKN2D//SH3BP4//UBE4B//GNAO1//BTG2//TRABD2B//FERMT3//FAF2//PRKAA1//VPS37D//GPR3//KCTD18//FGFRL1//OGT//CNIH2//GOSR2//CAMK2A//SEMA4B//SEMA4G//DPYS//GRIN2B//YWHAB//NPTX1//ANO3//MIF//RAB3B//IL4R//ZSWIM6//ABHD17B// |
| GO:0006935 | chemotaxis | Biological process | 14 | 594 | 169 | 17653 | 2.46191700037854 | 0.00175373240770669 | 0.388600905767914 | 2.75603667249914 | 0.0828402366863905 | EFNA3//GPC1//ARHGAP35//NCAM1//SPTBN5//PRKCQ//BDNF//CNTN2//UNC5A//MIF//SEMA4B//SEMA4G//ZSWIM6//RALBP1// |
| GO:0042330 | taxis | Biological process | 14 | 595 | 169 | 17653 | 2.45777932474765 | 0.00178125507597299 | 0.388600905767914 | 2.74927388505364 | 0.0828402366863905 | RALBP1//EFNA3//GPC1//ARHGAP35//NCAM1//SPTBN5//PRKCQ//BDNF//CNTN2//UNC5A//MIF//SEMA4B//SEMA4G//ZSWIM6// |
| GO:0060765 | regulation of androgen receptor signaling pathway | Biological process | 3 | 26 | 169 | 17653 | 12.0525716886664 | 0.00190605016900183 | 0.394675190918222 | 2.71986567251516 | 0.0177514792899408 | HEYL//SFRP1//EP300// |
| GO:0032989 | cellular component morphogenesis | Biological process | 21 | 1101 | 169 | 17653 | 1.99234155071506 | 0.00196140838248912 | 0.394675190918222 | 2.70743197298368 | 0.124260355029586 | CDH7//APBB1//EFNA3//GPC1//ARHGAP35//NCAM1//SPTBN5//PRKCQ//BDNF//CNTN2//UNC5A//RHOF//ENAM//EP300//SRF//FERMT3//CAMK2A//SEMA4B//SEMA4G//NPTX1//ZSWIM6// |
| GO:0120035 | regulation of plasma membrane bounded cell projection organization | Biological process | 14 | 602 | 169 | 17653 | 2.42920049539012 | 0.001984172185601 | 0.394675190918222 | 2.70242064265985 | 0.0828402366863905 | TWF1//BRK1//SFRP1//ARHGAP35//APBB1//CNTN2//SRF//BDNF//SEMA4B//SEMA4G//ZSWIM6//DYNLL1//ABHD17B//GRIN2B// |
| GO:0043270 | positive regulation of ion transport | Biological process | 8 | 240 | 169 | 17653 | 3.4818540433925 | 0.00222212810063216 | 0.421700182888955 | 2.65323090858414 | 0.0473372781065089 | P2RX2//GPD1L//SCN3B//CYP4F2//RAB3B//CAMK2A//MIF//CHRM1// |
| GO:0051641 | cellular localization | Biological process | 41 | 2761 | 169 | 17653 | 1.55113381867902 | 0.00224886242951318 | 0.421700182888955 | 2.64803711100754 | 0.242603550295858 | SPRN//NUP214//TWF1//SRSF3//YWHAB//ZDHHC22//GOSR2//TOMM40//ARFIP2//RABL3//KCNIP3//RAB3B//MLPH//CNIH2//SPTBN5//DYNLL1//VAPA//CPLX1//MGRN1//P2RX2//VPS37D//ABCA1//FAF2//VPS53//IL4R//CTDSPL2//CSPG5//CAMK2A//RIMS4//SH3BP4//TULP4//CNTN2//SCN3B//CHRM1//GRIN2B//ABHD17B//NUCB1//TP53INP2//PRKAA1//BDNF//GPD1L// |
| GO:0031344 | regulation of cell projection organization | Biological process | 14 | 612 | 169 | 17653 | 2.38950767683799 | 0.00230709844253901 | 0.421700182888955 | 2.63693387399241 | 0.0828402366863905 | TWF1//BRK1//SFRP1//ARHGAP35//APBB1//CNTN2//SRF//BDNF//SEMA4B//SEMA4G//ZSWIM6//DYNLL1//ABHD17B//GRIN2B// |
| GO:0006473 | protein acetylation | Biological process | 7 | 190 | 169 | 17653 | 3.84836499532856 | 0.00238769882176578 | 0.423488193769722 | 2.62202045488531 | 0.0414201183431953 | EP300//HAT1//FOXP3//APBB1//OGT//FAM161A//FOXO1// |
| GO:0009968 | negative regulation of signal transduction | Biological process | 23 | 1276 | 169 | 17653 | 1.88282076014172 | 0.00246899079812483 | 0.423488193769722 | 2.60748052867117 | 0.136094674556213 | MGRN1//YWHAB//SMPD1//TRABD2B//SFRP1//FAM89B//TOB1//CTDSPL2//SH3BP4//PRKAA1//FOXO1//ARHGAP35//GPATCH3//GPC1//MIF//PRKCQ//CGNL1//HEYL//GPD1L//PSMB8//PTPN18//CDKN2D//LYPD6// |
| GO:0046907 | intracellular transport | Biological process | 29 | 1758 | 169 | 17653 | 1.72310183034783 | 0.00253131117330836 | 0.423488193769722 | 2.59665446387217 | 0.171597633136095 | SPRN//NUP214//SRSF3//YWHAB//ZDHHC22//GOSR2//TOMM40//ARFIP2//RABL3//KCNIP3//RAB3B//MLPH//CNIH2//SPTBN5//DYNLL1//VAPA//MGRN1//CPLX1//VPS37D//ABCA1//FAF2//VPS53//IL4R//CTDSPL2//RIMS4//CHRM1//NUCB1//PRKAA1//CSPG5// |
| GO:2000516 | positive regulation of CD4-positive, alpha-beta T cell activation | Biological process | 3 | 29 | 169 | 17653 | 10.8057539277698 | 0.00262303432372301 | 0.423488193769722 | 2.5811960264108 | 0.0177514792899408 | FOXP3//IL4R//PRKCQ// |
| GO:0043269 | regulation of ion transport | Biological process | 14 | 622 | 169 | 17653 | 2.35109115470233 | 0.00267240269747655 | 0.423488193769722 | 2.57309809854886 | 0.0828402366863905 | P2RX2//GPD1L//SCN3B//CYP4F2//CHRM1//RAB3B//BEST1//GNAO1//CAMK2A//MIF//KCNIP3//SESTD1//OSR1//CNIH2// |
| GO:0032879 | regulation of localization | Biological process | 40 | 2700 | 169 | 17653 | 1.54749068595222 | 0.00269259091114861 | 0.423488193769722 | 2.56982962450402 | 0.236686390532544 | P2RX2//MIF//GPD1L//SCN3B//ABCA1//CPLX1//RAB3B//KIF2A//RHOF//SEMA4B//FAM89B//SEMA4G//FERMT3//SFRP1//SRF//CYP4F2//DYNLL1//CHRM1//IL4R//NUP214//CTDSPL2//CAMK2A//FOXP3//APBB1//BEST1//GNAO1//PRKAA1//YWHAB//KCNIP3//ABHD17B//ZSWIM6//CNIH2//NUCB1//TP53INP2//SHQ1//SESTD1//OSR1//BDNF//RIMS4//PAX8// |
| GO:0090066 | regulation of anatomical structure size | Biological process | 12 | 491 | 169 | 17653 | 2.55288687499247 | 0.00276686121924484 | 0.425279146039838 | 2.55801262373859 | 0.0710059171597633 | CHRM1//TWF1//ARHGAP35//BRK1//CNTN2//ARFIP2//SRF//BDNF//SEMA4B//SEMA4G//SPTBN5//RAB3B// |
| GO:0030032 | lamellipodium assembly | Biological process | 4 | 63 | 169 | 17653 | 6.63210293979525 | 0.00311236700693526 | 0.451552744292172 | 2.50690919712935 | 0.0236686390532544 | TWF1//BRK1//ARFIP2//ARHGEF6// |
| GO:0043967 | histone H4 acetylation | Biological process | 4 | 63 | 169 | 17653 | 6.63210293979525 | 0.00311236700693526 | 0.451552744292172 | 2.50690919712935 | 0.0236686390532544 | OGT//EP300//APBB1//HAT1// |
| GO:0010648 | negative regulation of cell communication | Biological process | 24 | 1380 | 169 | 17653 | 1.81661950090044 | 0.00315306138031857 | 0.451552744292172 | 2.50126757480421 | 0.142011834319527 | MGRN1//YWHAB//SMPD1//TRABD2B//SFRP1//FAM89B//TOB1//CTDSPL2//SH3BP4//PRKAA1//FOXO1//ARHGAP35//GPATCH3//GPC1//MIF//PRKCQ//CGNL1//SRF//HEYL//GPD1L//PSMB8//PTPN18//CDKN2D//LYPD6// |
| GO:0030182 | neuron differentiation | Biological process | 23 | 1303 | 169 | 17653 | 1.84380605521169 | 0.00320884722706273 | 0.451552744292172 | 2.4936509591348 | 0.136094674556213 | APBB1//EFNA3//GPC1//ARHGAP35//NCAM1//SPTBN5//PRKCQ//BDNF//CNTN2//UNC5A//SFRP1//TWF1//BTG2//UBE4B//GNAO1//SRF//VAPA//HEYL//CAMK2A//SEMA4B//SEMA4G//NPTX1//ZSWIM6// |
| GO:0023057 | negative regulation of signaling | Biological process | 24 | 1384 | 169 | 17653 | 1.81136915552211 | 0.00327163750854893 | 0.451552744292172 | 2.48523482136929 | 0.142011834319527 | MGRN1//YWHAB//SMPD1//TRABD2B//SFRP1//FAM89B//TOB1//CTDSPL2//SH3BP4//PRKAA1//FOXO1//ARHGAP35//GPATCH3//GPC1//MIF//PRKCQ//CGNL1//SRF//HEYL//GPD1L//PSMB8//PTPN18//CDKN2D//LYPD6// |
| GO:0035601 | protein deacylation | Biological process | 5 | 108 | 169 | 17653 | 4.8359083936007 | 0.00382306162170053 | 0.452070394660288 | 2.4175887017851 | 0.029585798816568 | ABHD17B//ARID4B//HDAC11//FOXP3//EP300// |
| GO:0051716 | cellular response to stimulus | Biological process | 90 | 7556 | 169 | 17653 | 1.24417759623607 | 0.00383102152481394 | 0.452070394660288 | 2.41668540790164 | 0.532544378698225 | GRIN2B//NCAM1//SPTBN5//PSMB8//YWHAB//CAMK2A//PRKAA1//COPS7B//CDKN2D//EP300//P2RX2//TRABD2B//NDP//GNAO1//RALBP1//PTPRT//AKAP10//CHRM1//ARHGAP35//KCNIP3//APBB1//IL4R//FGFRL1//SMPD1//OGT//VAPA//SH3BP5//BDNF//CARHSP1//PRKCQ//BTG2//POLR2E//FOXO1//MIF//CLEC1A//ABCA1//GNAZ//GPR153//SFRP1//ARHGEF6//GPR3//HEYL//EVC//ADAM11//FERMT3//OSR1//DDAH1//ARFIP2//RHOF//UBE4B//TOB1//CSPG5//MGRN1//BRK1//SH3BP4//FOXP3//FAF2//NPNT//FAM89B//CTDSPL2//CREB3L3//RABL3//RAB3B//KMT2D//GPX8//DYNLL1//GOSR2//UNC5A//PTPN18//PAX8//GPATCH3//GPC1//SRF//CRIM1//EFNA3//CGNL1//CNTN2//SP5//SEMA4B//SEMA4G//ZNF385B//CASP7//GPD1L//LMO2//BSN//LYPD6//NUP214//SRSF3//CNIH2//NUDT16// |
| GO:0007584 | response to nutrient | Biological process | 7 | 207 | 169 | 17653 | 3.5323156961953 | 0.00383817963316018 | 0.452070394660288 | 2.41587470342425 | 0.0414201183431953 | P2RX2//CDKN2D//SRF//SFRP1//ABCA1//OGT//HAT1// |
| GO:0042758 | long-chain fatty acid catabolic process | Biological process | 2 | 10 | 169 | 17653 | 20.891124260355 | 0.0038982561838201 | 0.452070394660288 | 2.40912962351406 | 0.0118343195266272 | CYP4F3//CYP4F2// |
| GO:1901569 | fatty acid derivative catabolic process | Biological process | 2 | 10 | 169 | 17653 | 20.891124260355 | 0.0038982561838201 | 0.452070394660288 | 2.40912962351406 | 0.0118343195266272 | CYP4F3//CYP4F2// |
| GO:1902950 | regulation of dendritic spine maintenance | Biological process | 2 | 10 | 169 | 17653 | 20.891124260355 | 0.0038982561838201 | 0.452070394660288 | 2.40912962351406 | 0.0118343195266272 | GRIN2B//ABHD17B// |
| GO:0098732 | macromolecule deacylation | Biological process | 5 | 109 | 169 | 17653 | 4.79154226154932 | 0.0039764596079589 | 0.452070394660288 | 2.40050342466091 | 0.029585798816568 | ABHD17B//ARID4B//HDAC11//FOXP3//EP300// |
| GO:0007015 | actin filament organization | Biological process | 10 | 388 | 169 | 17653 | 2.69215518819008 | 0.00425623682638691 | 0.452070394660288 | 2.37097421493963 | 0.0591715976331361 | RHOF//TWF1//MICAL2//ARHGAP35//BRK1//ARFIP2//SRF//SFRP1//SPTBN5//CGNL1// |
| GO:0048519 | negative regulation of biological process | Biological process | 67 | 5303 | 169 | 17653 | 1.31972970530246 | 0.00469485705800167 | 0.452070394660288 | 2.32837762594657 | 0.396449704142012 | OSR1//EP300//FOXO1//HEYL//ARHGAP35//KCNIP3//APBB1//SP5//FOXP3//BTG2//MIF//TWF1//BAHD1//KMT2D//HAT1//NUDT16//CDKN2D//PRKAA1//SFRP1//TOB1//DDAH1//SH3BP4//FGFRL1//SRF//PAX8//ABCA1//BDNF//PPP1R26//CRIM1//PSMB8//MGRN1//YWHAB//EFNA3//PURA//BTAF1//MEX3A//SMPD1//TRABD2B//FAM89B//CTDSPL2//OGT//CYP4F2//GPATCH3//GPD1L//GPC1//DYNLL1//CARHSP1//NUP214//VAPA//IL4R//CNTN2//PRKCQ//SEMA4B//SEMA4G//CGNL1//SPTBN5//GNAO1//POLR2E//SH3BP5//PTPN18//CNIH2//ABHD17B//GRIN2B//TP53INP2//SESTD1//SHQ1//LYPD6// |
| GO:0060211 | regulation of nuclear-transcribed mRNA poly(A) tail shortening | Biological process | 2 | 11 | 169 | 17653 | 18.9919311457773 | 0.00473466466713258 | 0.452070394660288 | 2.32471077450042 | 0.0118343195266272 | TOB1//BTG2// |
| GO:0060213 | positive regulation of nuclear-transcribed mRNA poly(A) tail shortening | Biological process | 2 | 11 | 169 | 17653 | 18.9919311457773 | 0.00473466466713258 | 0.452070394660288 | 2.32471077450042 | 0.0118343195266272 | TOB1//BTG2// |
| GO:0051179 | localization | Biological process | 79 | 6497 | 169 | 17653 | 1.27012376217335 | 0.00492724412255339 | 0.452070394660288 | 2.30739591987419 | 0.467455621301775 | SPRN//NUP214//SEMA4B//SEMA4G//SRF//ISLR//FERMT3//TWF1//AKAP10//SRSF3//YWHAB//ZDHHC22//GOSR2//TOMM40//SLC26A1//P2RX2//SCN3B//CAMK2A//BEST1//SLC39A2//RALBP1//ABCA1//PCTP//ANO3//ARFIP2//RABL3//KCNIP3//RAB3B//MLPH//CPLX1//CNIH2//SPTBN5//DYNLL1//VAPA//KIF2A//SH3BP4//VPS53//MGRN1//ATP1B4//MIF//GPD1L//VPS37D//AVL9//ARHGAP35//RHOF//CNTN2//FAM89B//SFRP1//FAF2//CYP4F2//BSN//BRK1//CHRM1//IL4R//ARMC8//NPNT//CTDSPL2//CSPG5//RIMS4//FOXP3//APBB1//MOGAT2//GPC1//GNAO1//PSMB8//PRKCQ//PRKAA1//TULP4//GRIN2B//ABHD17B//NDUFA4//ZSWIM6//NUCB1//TP53INP2//SHQ1//SESTD1//OSR1//BDNF//PAX8// |
| GO:0051259 | protein complex oligomerization | Biological process | 12 | 528 | 169 | 17653 | 2.37399139322216 | 0.0049349112550004 | 0.452070394660288 | 2.30672065288496 | 0.0710059171597633 | EP300//TRABD2B//KCTD18//P2RX2//SPTBN5//FGFRL1//DPYS//GRIN2B//PRKAA1//YWHAB//MIF//BRK1// |
| GO:1902532 | negative regulation of intracellular signal transduction | Biological process | 12 | 528 | 169 | 17653 | 2.37399139322216 | 0.0049349112550004 | 0.452070394660288 | 2.30672065288496 | 0.0710059171597633 | SH3BP4//PRKAA1//FOXO1//ARHGAP35//GPATCH3//SMPD1//SFRP1//MIF//MGRN1//CGNL1//GPD1L//CDKN2D// |
| GO:0007268 | chemical synaptic transmission | Biological process | 14 | 667 | 169 | 17653 | 2.19247181143156 | 0.00495584106400585 | 0.452070394660288 | 2.30488263008695 | 0.0828402366863905 | CPLX1//CHRM1//P2RX2//CNIH2//CAMK2A//RIMS4//GRIN2B//CNTN2//CSPG5//BDNF//RAB3B//SRF//NPTX1//BSN// |
| GO:0098916 | anterograde trans-synaptic signaling | Biological process | 14 | 667 | 169 | 17653 | 2.19247181143156 | 0.00495584106400585 | 0.452070394660288 | 2.30488263008695 | 0.0828402366863905 | CPLX1//GRIN2B//NPTX1//BSN//CHRM1//P2RX2//CNIH2//CAMK2A//RIMS4//CNTN2//CSPG5//BDNF//RAB3B//SRF// |
| GO:0001558 | regulation of cell growth | Biological process | 10 | 397 | 169 | 17653 | 2.63112396226134 | 0.00499000221939662 | 0.452070394660288 | 2.30189926121599 | 0.0591715976331361 | SFRP1//CDKN2D//SH3BP4//APBB1//SRF//BDNF//SEMA4B//SEMA4G//CRIM1//PRKCQ// |
| GO:0050804 | modulation of chemical synaptic transmission | Biological process | 9 | 335 | 169 | 17653 | 2.80627042303277 | 0.00505282292608871 | 0.452070394660288 | 2.29646592115026 | 0.0532544378698225 | CPLX1//CAMK2A//RIMS4//GRIN2B//CNTN2//RAB3B//SRF//BDNF//CSPG5// |
| GO:0099177 | regulation of trans-synaptic signaling | Biological process | 9 | 335 | 169 | 17653 | 2.80627042303277 | 0.00505282292608871 | 0.452070394660288 | 2.29646592115026 | 0.0532544378698225 | CPLX1//CAMK2A//RIMS4//GRIN2B//CNTN2//CSPG5//BDNF//RAB3B//SRF// |
| GO:0034613 | cellular protein localization | Biological process | 28 | 1762 | 169 | 17653 | 1.65990771648678 | 0.00508441072573662 | 0.452070394660288 | 2.2937593737915 | 0.165680473372781 | SPRN//NUP214//TWF1//SRSF3//YWHAB//ZDHHC22//GOSR2//TOMM40//ARFIP2//RABL3//KCNIP3//RAB3B//MLPH//FAF2//CTDSPL2//SH3BP4//TULP4//VAPA//CNTN2//SCN3B//CHRM1//DYNLL1//ABHD17B//NUCB1//TP53INP2//PRKAA1//BDNF//GPD1L// |
| GO:0010646 | regulation of cell communication | Biological process | 48 | 3522 | 169 | 17653 | 1.42358598026269 | 0.00509214401953585 | 0.452070394660288 | 2.29309932182804 | 0.284023668639053 | PRKAA1//TOB1//CSPG5//MIF//NDP//BDNF//MGRN1//YWHAB//SMPD1//FOXP3//SFRP1//TRABD2B//NPNT//FAM89B//CTDSPL2//SH3BP4//ABCA1//FOXO1//KMT2D//OGT//ARHGEF6//ARHGAP35//DYNLL1//GPATCH3//GPC1//VAPA//EP300//EVC//PRKCQ//CPLX1//CAMK2A//RIMS4//GRIN2B//CNTN2//P2RX2//RALBP1//RHOF//CGNL1//RAB3B//SRF//HEYL//GPD1L//PSMB8//LYPD6//PTPN18//CDKN2D//CNIH2//PAX8// |
| GO:0099537 | trans-synaptic signaling | Biological process | 14 | 670 | 169 | 17653 | 2.18265477346993 | 0.00515177246498743 | 0.452070394660288 | 2.28804332642902 | 0.0828402366863905 | CPLX1//GRIN2B//NPTX1//BSN//CHRM1//P2RX2//CNIH2//CAMK2A//RIMS4//CNTN2//CSPG5//BDNF//RAB3B//SRF// |
| GO:0007519 | skeletal muscle tissue development | Biological process | 6 | 165 | 169 | 17653 | 3.79838622915546 | 0.00515637973220008 | 0.452070394660288 | 2.28765510731427 | 0.0355029585798817 | HEYL//BTG2//PRKAA1//P2RX2//GPC1//EP300// |
| GO:0099536 | synaptic signaling | Biological process | 14 | 671 | 169 | 17653 | 2.17940193476133 | 0.00521845919574105 | 0.452070394660288 | 2.28245770802576 | 0.0828402366863905 | CPLX1//GRIN2B//NPTX1//BSN//CHRM1//P2RX2//CNIH2//CAMK2A//RIMS4//CNTN2//CSPG5//BDNF//RAB3B//SRF// |
| GO:1902667 | regulation of axon guidance | Biological process | 3 | 37 | 169 | 17653 | 8.46937470014393 | 0.00527451927844636 | 0.452070394660288 | 2.27781711597941 | 0.0177514792899408 | SEMA4B//SEMA4G//ZSWIM6// |
| GO:0051254 | positive regulation of RNA metabolic process | Biological process | 25 | 1523 | 169 | 17653 | 1.71463593732395 | 0.0054463726662354 | 0.452070394660288 | 2.26389264552409 | 0.14792899408284 | SRF//EP300//HEYL//MICAL2//FOXO1//APBB1//NDP//FOXP3//ATXN7L3//TP53INP2//GPATCH3//SFRP1//PAX8//OSR1//ATP1B4//MCIDAS//LMO2//ARID4B//KMT2D//CREB3L3//OGT//TOB1//BTG2//PRKAA1//NPNT// |
| GO:0070727 | cellular macromolecule localization | Biological process | 28 | 1772 | 169 | 17653 | 1.65054029145017 | 0.0054908001570732 | 0.452070394660288 | 2.2603643625663 | 0.165680473372781 | SPRN//NUP214//TWF1//SRSF3//YWHAB//ZDHHC22//GOSR2//TOMM40//ARFIP2//RABL3//KCNIP3//RAB3B//MLPH//FAF2//CTDSPL2//SH3BP4//TULP4//VAPA//CNTN2//SCN3B//CHRM1//DYNLL1//ABHD17B//NUCB1//TP53INP2//PRKAA1//BDNF//GPD1L// |
| GO:0043627 | response to estrogen | Biological process | 4 | 74 | 169 | 17653 | 5.64624980009595 | 0.00554391586703144 | 0.452070394660288 | 2.2561833690819 | 0.0236686390532544 | SFRP1//EP300//IL4R//KMT2D// |
| GO:0006464 | cellular protein modification process | Biological process | 55 | 4192 | 169 | 17653 | 1.37048167261394 | 0.00563701141375136 | 0.452070394660288 | 2.2489510858248 | 0.325443786982249 | CDKN2D//GRIN2B//NCAM1//SPTBN5//PSMB8//YWHAB//CAMK2A//PRKAA1//UBE4B//MGRN1//COPS7B//ABHD17B//TULP4//STK35//PTPRT//PTPN18//CTDSPL2//LHPP//EP300//FOXO1//HAT1//BTG2//OGT//ABCA1//SPRN//PYURF//ARHGEF6//ATXN7L3//B4GALT5//ARID4B//HDAC11//USP54//NUP214//PRKCQ//OSR1//RAB3B//ZDHHC22//ST6GALNAC6//FOXP3//PPP1R26//GPD1L//MIF//SMPD1//SFRP1//TWF1//UBXN2B//ENAM//NUCB1//APBB1//KMT2D//SH3BP5//NPNT//FAM161A//TRABD2B//CHRM1// |
| GO:0036211 | protein modification process | Biological process | 55 | 4192 | 169 | 17653 | 1.37048167261394 | 0.00563701141375136 | 0.452070394660288 | 2.2489510858248 | 0.325443786982249 | CDKN2D//GRIN2B//NCAM1//SPTBN5//PSMB8//YWHAB//CAMK2A//PRKAA1//UBE4B//MGRN1//COPS7B//ABHD17B//TULP4//CHRM1//STK35//PTPRT//PTPN18//CTDSPL2//LHPP//EP300//FOXO1//HAT1//BTG2//OGT//ABCA1//SPRN//PYURF//ARHGEF6//ATXN7L3//B4GALT5//ARID4B//HDAC11//USP54//NUP214//PRKCQ//OSR1//RAB3B//ZDHHC22//ST6GALNAC6//FOXP3//PPP1R26//GPD1L//MIF//SMPD1//SFRP1//TWF1//UBXN2B//ENAM//NUCB1//APBB1//KMT2D//SH3BP5//NPNT//FAM161A//TRABD2B// |
| GO:0033631 | cell-cell adhesion mediated by integrin | Biological process | 2 | 12 | 169 | 17653 | 17.4092702169625 | 0.00564600476862187 | 0.452070394660288 | 2.24825875938334 | 0.0118343195266272 | FERMT3//NPNT// |
| GO:0043116 | negative regulation of vascular permeability | Biological process | 2 | 12 | 169 | 17653 | 17.4092702169625 | 0.00564600476862187 | 0.452070394660288 | 2.24825875938334 | 0.0118343195266272 | DDAH1//ARHGAP35// |
| GO:0060766 | negative regulation of androgen receptor signaling pathway | Biological process | 2 | 12 | 169 | 17653 | 17.4092702169625 | 0.00564600476862187 | 0.452070394660288 | 2.24825875938334 | 0.0118343195266272 | HEYL//SFRP1// |
| GO:2000232 | regulation of rRNA processing | Biological process | 2 | 12 | 169 | 17653 | 17.4092702169625 | 0.00564600476862187 | 0.452070394660288 | 2.24825875938334 | 0.0118343195266272 | NUDT16//SHQ1// |
| GO:0051649 | establishment of localization in cell | Biological process | 32 | 2117 | 169 | 17653 | 1.57892294834993 | 0.00573988494217508 | 0.452070394660288 | 2.24109681308622 | 0.189349112426035 | SPRN//NUP214//SRSF3//YWHAB//ZDHHC22//GOSR2//TOMM40//ARFIP2//RABL3//KCNIP3//RAB3B//MLPH//CNIH2//SPTBN5//DYNLL1//VAPA//CPLX1//MGRN1//P2RX2//VPS37D//ABCA1//FAF2//VPS53//IL4R//CTDSPL2//CSPG5//CAMK2A//RIMS4//CHRM1//GRIN2B//NUCB1//PRKAA1// |
| GO:0033143 | regulation of intracellular steroid hormone receptor signaling pathway | Biological process | 4 | 75 | 169 | 17653 | 5.57096646942801 | 0.00581333014347121 | 0.452070394660288 | 2.235575012427 | 0.0236686390532544 | KMT2D//EP300//HEYL//SFRP1// |
| GO:0043412 | macromolecule modification | Biological process | 57 | 4389 | 169 | 17653 | 1.35656651041266 | 0.00587400907733282 | 0.452070394660288 | 2.23106538668068 | 0.337278106508876 | CDKN2D//THUMPD1//GRIN2B//NCAM1//SPTBN5//PSMB8//YWHAB//CAMK2A//PRKAA1//UBE4B//MGRN1//COPS7B//ABHD17B//TULP4//NUDT16//CHRM1//STK35//PTPRT//PTPN18//CTDSPL2//LHPP//EP300//FOXO1//HAT1//BTG2//OGT//ABCA1//SPRN//PYURF//ARHGEF6//ATXN7L3//B4GALT5//ARID4B//HDAC11//USP54//NUP214//PRKCQ//OSR1//RAB3B//ZDHHC22//ST6GALNAC6//FOXP3//PPP1R26//GPD1L//MIF//SMPD1//SFRP1//TWF1//UBXN2B//ENAM//NUCB1//APBB1//KMT2D//SH3BP5//NPNT//FAM161A//TRABD2B// |
| GO:0046434 | organophosphate catabolic process | Biological process | 7 | 224 | 169 | 17653 | 3.26423816568047 | 0.00587772173123405 | 0.452070394660288 | 2.23079097856456 | 0.0414201183431953 | NUP214//OGT//SMPD1//PLA2G15//NUDT16//PRKAA1//GPD1L// |
| GO:0048518 | positive regulation of biological process | Biological process | 72 | 5841 | 169 | 17653 | 1.28758855225609 | 0.00589777965501082 | 0.452070394660288 | 2.2293114570132 | 0.42603550295858 | PRKAA1//SFRP1//EP300//PSMB8//IL4R//FOXP3//CREB3L3//SRF//BTG2//HEYL//CHRM1//NUDT16//BRK1//PURA//KMT2D//CASP7//NPNT//PYURF//TOB1//SCN3B//FOXO1//SH3BP4//TP53INP2//P2RX2//TWF1//OSR1//CAMK2A//MICAL2//MIF//GPD1L//ABCA1//CNTN2//ARHGAP35//APBB1//NDP//ATXN7L3//GPATCH3//PAX8//PRKCQ//SEMA4B//FAM89B//SEMA4G//FERMT3//BDNF//CYP4F2//TRABD2B//POLR3H//POLR2E//ARFIP2//SMPD1//DYNLL1//SHQ1//ARHGEF6//VAPA//DDAH1//OGT//EVC//ATP1B4//MCIDAS//LMO2//ARID4B//CTDSPL2//RIMS4//RAB3B//GRIN2B//ENAM//YWHAB//FAM161A//EFNA3//ABHD17B//DENND6A//GPC1// |
| GO:0030308 | negative regulation of cell growth | Biological process | 6 | 170 | 169 | 17653 | 3.68666898712148 | 0.00594917420150313 | 0.452070394660288 | 2.22554331403959 | 0.0355029585798817 | SEMA4B//SEMA4G//CDKN2D//SH3BP4//APBB1//SFRP1// |
| GO:0016049 | cell growth | Biological process | 11 | 477 | 169 | 17653 | 2.40882984134073 | 0.006311203270554 | 0.46569617972635 | 2.19988783189015 | 0.0650887573964497 | KMT2D//CRIM1//PRKCQ//SFRP1//CDKN2D//SH3BP4//APBB1//SRF//BDNF//SEMA4B//SEMA4G// |
| GO:0048468 | cell development | Biological process | 31 | 2045 | 169 | 17653 | 1.58343484613938 | 0.00631670696255128 | 0.46569617972635 | 2.19950926993148 | 0.183431952662722 | KMT2D//SEMA4B//SEMA4G//DYNLL1//APBB1//EFNA3//GPC1//ARHGAP35//NCAM1//SPTBN5//PRKCQ//BDNF//CNTN2//UNC5A//SFRP1//TWF1//HDAC11//HEYL//BTG2//EP300//SRF//UBE4B//GNAO1//VAPA//FERMT3//P2RX2//CAMK2A//NPTX1//OSR1//ARID4B//ZSWIM6// |
| GO:0009653 | anatomical structure morphogenesis | Biological process | 38 | 2656 | 169 | 17653 | 1.49447048549227 | 0.0063350655825557 | 0.46569617972635 | 2.19824888480665 | 0.224852071005917 | CDH7//SRF//NPNT//PAX8//EP300//ARHGAP35//MICAL2//HEYL//FGFRL1//UBE4B//APBB1//EFNA3//GPC1//NCAM1//SPTBN5//PRKCQ//BDNF//CNTN2//UNC5A//RHOF//SFRP1//ENAM//FERMT3//OSR1//SP5//TMIE//DDAH1//HOXC9//CAMK2A//SEMA4B//SEMA4G//PSMB8//NPTX1//NDP//FOXO1//IL4R//ZSWIM6//FOXP3// |
| GO:0007166 | cell surface receptor signaling pathway | Biological process | 42 | 3016 | 169 | 17653 | 1.45462072104594 | 0.00641018954596897 | 0.466151740853636 | 2.19312912842949 | 0.248520710059172 | EP300//PSMB8//TRABD2B//NDP//PRKAA1//GNAO1//CAMK2A//PTPRT//BDNF//GRIN2B//HEYL//EVC//ADAM11//FERMT3//FOXO1//FGFRL1//POLR2E//IL4R//FOXP3//PRKCQ//SFRP1//TOB1//NPNT//FAM89B//CTDSPL2//MIF//P2RX2//UNC5A//ABCA1//BRK1//PTPN18//GPC1//MGRN1//OGT//CRIM1//EFNA3//CNTN2//NCAM1//SEMA4B//SEMA4G//CNIH2//CLEC1A// |
| GO:0072182 | regulation of nephron tubule epithelial cell differentiation | Biological process | 2 | 13 | 169 | 17653 | 16.0700955848885 | 0.00663078239512773 | 0.477063631257966 | 2.178435224259 | 0.0118343195266272 | OSR1//PAX8// |
| GO:0023051 | regulation of signaling | Biological process | 48 | 3575 | 169 | 17653 | 1.40248106922663 | 0.00681106476395847 | 0.484876115775275 | 2.16678499014816 | 0.284023668639053 | PRKAA1//TOB1//CSPG5//MIF//NDP//BDNF//MGRN1//YWHAB//SMPD1//FOXP3//SFRP1//TRABD2B//NPNT//FAM89B//CTDSPL2//SH3BP4//ABCA1//FOXO1//KMT2D//OGT//ARHGEF6//ARHGAP35//DYNLL1//GPATCH3//GPC1//VAPA//EP300//EVC//PRKCQ//CPLX1//CAMK2A//RIMS4//GRIN2B//CNTN2//P2RX2//RALBP1//RHOF//CGNL1//RAB3B//SRF//HEYL//GPD1L//PSMB8//LYPD6//PTPN18//CDKN2D//CNIH2//PAX8// |
| GO:0048583 | regulation of response to stimulus | Biological process | 55 | 4243 | 169 | 17653 | 1.35400876068763 | 0.00729807847624165 | 0.514134424321065 | 2.13679147097747 | 0.325443786982249 | PRKAA1//EP300//PSMB8//CREB3L3//FOXP3//TOB1//CSPG5//MIF//NDP//BDNF//TWF1//MGRN1//YWHAB//PRKCQ//SMPD1//SFRP1//TRABD2B//NPNT//FAM89B//CTDSPL2//SH3BP4//ABCA1//FOXO1//KMT2D//OGT//ARHGEF6//ARHGAP35//DYNLL1//BRK1//GPATCH3//GPC1//VAPA//IL4R//APBB1//EVC//SEMA4B//SEMA4G//P2RX2//RALBP1//RHOF//CGNL1//RAB3B//CNTN2//HEYL//GPD1L//LYPD6//NUP214//CAMK2A//DDAH1//PTPN18//CDKN2D//ZSWIM6//OSR1//CNIH2//NUDT16// |
| GO:0097581 | lamellipodium organization | Biological process | 4 | 81 | 169 | 17653 | 5.15830228650741 | 0.0076142529201033 | 0.514759711758177 | 2.1183727014669 | 0.0236686390532544 | TWF1//BRK1//ARFIP2//ARHGEF6// |
| GO:0030950 | establishment or maintenance of actin cytoskeleton polarity | Biological process | 2 | 14 | 169 | 17653 | 14.9222316145393 | 0.00768752489835515 | 0.514759711758177 | 2.11421346462131 | 0.0118343195266272 | ARHGAP35//RHOF// |
| GO:0045623 | negative regulation of T-helper cell differentiation | Biological process | 2 | 14 | 169 | 17653 | 14.9222316145393 | 0.00768752489835515 | 0.514759711758177 | 2.11421346462131 | 0.0118343195266272 | IL4R//FOXP3// |
| GO:0090069 | regulation of ribosome biogenesis | Biological process | 2 | 14 | 169 | 17653 | 14.9222316145393 | 0.00768752489835515 | 0.514759711758177 | 2.11421346462131 | 0.0118343195266272 | NUDT16//SHQ1// |
| GO:2000316 | regulation of T-helper 17 type immune response | Biological process | 2 | 14 | 169 | 17653 | 14.9222316145393 | 0.00768752489835515 | 0.514759711758177 | 2.11421346462131 | 0.0118343195266272 | PRKCQ//FOXP3// |
| GO:0051049 | regulation of transport | Biological process | 28 | 1821 | 169 | 17653 | 1.60612707108715 | 0.00789885676918418 | 0.52372517970581 | 2.10243576121123 | 0.165680473372781 | P2RX2//GPD1L//SCN3B//ABCA1//CPLX1//RAB3B//CYP4F2//DYNLL1//CHRM1//IL4R//SFRP1//NUP214//CTDSPL2//CAMK2A//FOXP3//APBB1//MIF//BEST1//GNAO1//PRKAA1//YWHAB//KCNIP3//NUCB1//CNIH2//SESTD1//OSR1//RIMS4//PAX8// |
| GO:0061013 | regulation of mRNA catabolic process | Biological process | 6 | 181 | 169 | 17653 | 3.46261728072183 | 0.00800131763201278 | 0.525368069371868 | 2.09683848885901 | 0.0355029585798817 | CARHSP1//PSMB8//YWHAB//NUP214//TOB1//BTG2// |
| GO:0007267 | cell-cell signaling | Biological process | 26 | 1658 | 169 | 17653 | 1.63802542451517 | 0.00822043155227468 | 0.529494781156951 | 2.08510538247797 | 0.153846153846154 | TRABD2B//NDP//PRKAA1//GNAO1//CAMK2A//CPLX1//GRIN2B//NPTX1//BSN//CHRM1//P2RX2//FOXP3//SFRP1//CNIH2//DYNLL1//RIMS4//CNTN2//CSPG5//BDNF//RAB3B//PSMB8//SRF//SCN3B//FOXO1//PAX8//EFNA3// |
| GO:0045935 | positive regulation of nucleobase-containing compound metabolic process | Biological process | 27 | 1742 | 169 | 17653 | 1.61900216713429 | 0.00822075292347773 | 0.529494781156951 | 2.08508840441409 | 0.159763313609467 | SRF//EP300//HEYL//MICAL2//FOXO1//APBB1//NDP//FOXP3//ATXN7L3//TP53INP2//GPATCH3//SFRP1//PAX8//PRKCQ//PRKAA1//OSR1//ATP1B4//MCIDAS//LMO2//ARID4B//KMT2D//CREB3L3//OGT//TOB1//BTG2//NPNT//NUDT16// |
| GO:0010959 | regulation of metal ion transport | Biological process | 9 | 363 | 169 | 17653 | 2.589808792606 | 0.00837933136861388 | 0.534617151376752 | 2.07679063465215 | 0.0532544378698225 | P2RX2//GPD1L//SCN3B//BEST1//GNAO1//CAMK2A//KCNIP3//SESTD1//OSR1// |
| GO:0061061 | muscle structure development | Biological process | 13 | 640 | 169 | 17653 | 2.12175480769231 | 0.00869825794234181 | 0.541729093193857 | 2.06056771770142 | 0.0769230769230769 | UBE4B//SRF//EVC//EP300//BDNF//HEYL//BTG2//PRKAA1//P2RX2//FGFRL1//IL4R//NPNT//GPC1// |
| GO:0032305 | positive regulation of icosanoid secretion | Biological process | 2 | 15 | 169 | 17653 | 13.92741617357 | 0.0088147808051938 | 0.541729093193857 | 2.05478848271558 | 0.0118343195266272 | MIF//CYP4F2// |
| GO:0043371 | negative regulation of CD4-positive, alpha-beta T cell differentiation | Biological process | 2 | 15 | 169 | 17653 | 13.92741617357 | 0.0088147808051938 | 0.541729093193857 | 2.05478848271558 | 0.0118343195266272 | IL4R//FOXP3// |
| GO:0072160 | nephron tubule epithelial cell differentiation | Biological process | 2 | 15 | 169 | 17653 | 13.92741617357 | 0.0088147808051938 | 0.541729093193857 | 2.05478848271558 | 0.0118343195266272 | OSR1//PAX8// |
| GO:0006470 | protein dephosphorylation | Biological process | 8 | 303 | 169 | 17653 | 2.75790419278614 | 0.00889130997257402 | 0.541729093193857 | 2.05103424893432 | 0.0473372781065089 | PPP1R26//SMPD1//YWHAB//PTPRT//PTPN18//UBXN2B//CTDSPL2//LHPP// |
| GO:2000514 | regulation of CD4-positive, alpha-beta T cell activation | Biological process | 3 | 45 | 169 | 17653 | 6.96370808678501 | 0.00911089054673881 | 0.545600938719608 | 2.04043917070736 | 0.0177514792899408 | FOXP3//IL4R//PRKCQ// |
| GO:0045620 | negative regulation of lymphocyte differentiation | Biological process | 3 | 46 | 169 | 17653 | 6.81232312837664 | 0.0096790711283099 | 0.545600938719608 | 2.01416631864259 | 0.0177514792899408 | SFRP1//IL4R//FOXP3// |
| GO:0007167 | enzyme linked receptor protein signaling pathway | Biological process | 18 | 1028 | 169 | 17653 | 1.82898947804665 | 0.00972093859923112 | 0.545600938719608 | 2.01229180001333 | 0.106508875739645 | PTPRT//BDNF//FOXO1//FGFRL1//POLR2E//TOB1//NPNT//FAM89B//CTDSPL2//SFRP1//PTPN18//GPC1//OGT//PRKCQ//CRIM1//BRK1//EFNA3//GRIN2B// |
| GO:0006672 | ceramide metabolic process | Biological process | 4 | 87 | 169 | 17653 | 4.80255730123104 | 0.00974848878241954 | 0.545600938719608 | 2.01106270371937 | 0.0236686390532544 | ST6GALNAC6//PRKAA1//SMPD1//PLA2G15// |
| GO:0071495 | cellular response to endogenous stimulus | Biological process | 22 | 1350 | 169 | 17653 | 1.70223975454745 | 0.00983636402852218 | 0.545600938719608 | 2.00716540707312 | 0.130177514792899 | CHRM1//FOXO1//FGFRL1//POLR2E//SH3BP4//TOB1//NPNT//FAM89B//CTDSPL2//SFRP1//KMT2D//CAMK2A//BDNF//GPC1//OGT//PRKCQ//EP300//HEYL//PAX8//PRKAA1//CASP7//LMO2// |
| GO:0034315 | regulation of Arp2/3 complex-mediated actin nucleation | Biological process | 2 | 16 | 169 | 17653 | 13.0569526627219 | 0.0100111195511873 | 0.545600938719608 | 1.99951735231939 | 0.0118343195266272 | BRK1//ARFIP2// |
| GO:0035855 | megakaryocyte development | Biological process | 2 | 16 | 169 | 17653 | 13.0569526627219 | 0.0100111195511873 | 0.545600938719608 | 1.99951735231939 | 0.0118343195266272 | EP300//SRF// |
| GO:0070166 | enamel mineralization | Biological process | 2 | 16 | 169 | 17653 | 13.0569526627219 | 0.0100111195511873 | 0.545600938719608 | 1.99951735231939 | 0.0118343195266272 | ENAM//FOXO1// |
| GO:1900151 | regulation of nuclear-transcribed mRNA catabolic process, deadenylation-dependent decay | Biological process | 2 | 16 | 169 | 17653 | 13.0569526627219 | 0.0100111195511873 | 0.545600938719608 | 1.99951735231939 | 0.0118343195266272 | TOB1//BTG2// |
| GO:1900153 | positive regulation of nuclear-transcribed mRNA catabolic process, deadenylation-dependent decay | Biological process | 2 | 16 | 169 | 17653 | 13.0569526627219 | 0.0100111195511873 | 0.545600938719608 | 1.99951735231939 | 0.0118343195266272 | TOB1//BTG2// |
| GO:2000193 | positive regulation of fatty acid transport | Biological process | 2 | 16 | 169 | 17653 | 13.0569526627219 | 0.0100111195511873 | 0.545600938719608 | 1.99951735231939 | 0.0118343195266272 | CYP4F2//MIF// |
| GO:0048699 | generation of neurons | Biological process | 23 | 1437 | 169 | 17653 | 1.67187146133669 | 0.0102234471648385 | 0.545600938719608 | 1.99040264312068 | 0.136094674556213 | SRF//APBB1//EFNA3//GPC1//ARHGAP35//NCAM1//SPTBN5//PRKCQ//BDNF//CNTN2//UNC5A//SFRP1//TWF1//BTG2//UBE4B//GNAO1//VAPA//HEYL//CAMK2A//SEMA4B//SEMA4G//NPTX1//ZSWIM6// |
| GO:0009719 | response to endogenous stimulus | Biological process | 25 | 1606 | 169 | 17653 | 1.62602150220696 | 0.0104178731331778 | 0.545600938719608 | 1.98222093562441 | 0.14792899408284 | CHRM1//FOXO1//FGFRL1//POLR2E//SRF//SH3BP4//TOB1//NPNT//FAM89B//CTDSPL2//SFRP1//OGT//KMT2D//CAMK2A//BDNF//GPC1//BTG2//PRKCQ//EP300//HEYL//PAX8//PRKAA1//ABCA1//CASP7//LMO2// |
| GO:0006811 | ion transport | Biological process | 25 | 1610 | 169 | 17653 | 1.62198169723253 | 0.0107294359007709 | 0.545600938719608 | 1.96942311043129 | 0.14792899408284 | P2RX2//SCN3B//CAMK2A//SLC26A1//BEST1//SLC39A2//ATP1B4//GPD1L//CPLX1//PCTP//CYP4F2//ABCA1//ANO3//CHRM1//RAB3B//GNAO1//MIF//KCNIP3//GRIN2B//RALBP1//NDUFA4//SESTD1//OSR1//CNIH2//TOMM40// |
| GO:0051588 | regulation of neurotransmitter transport | Biological process | 4 | 90 | 169 | 17653 | 4.64247205785667 | 0.0109475052612307 | 0.545600938719608 | 1.96068483741943 | 0.0236686390532544 | CPLX1//CAMK2A//RAB3B//RIMS4// |
| GO:0072073 | kidney epithelium development | Biological process | 5 | 140 | 169 | 17653 | 3.73055790363483 | 0.0112170908346244 | 0.545600938719608 | 1.95011976324848 | 0.029585798816568 | OSR1//NPNT//SFRP1//PAX8//HEYL// |
| GO:0022607 | cellular component assembly | Biological process | 40 | 2934 | 169 | 17653 | 1.42407118339162 | 0.0112349089954759 | 0.545600938719608 | 1.94943044108399 | 0.236686390532544 | UBXN2B//TP53INP2//SHQ1//RHOF//TWF1//ASF1B//HAT1//CDH7//BDNF//BSN//MCIDAS//FAM161A//DYNLL1//BRK1//ISCU//ARFIP2//ARHGEF6//BAHD1//GPC1//EP300//TRABD2B//FERMT3//ABCA1//VPS37D//KCTD18//P2RX2//SPTBN5//FGFRL1//SRF//ARHGAP35//CNIH2//GOSR2//DPYS//GRIN2B//PRKAA1//YWHAB//SFRP1//MIF//KIF2A//KMT2D// |
| GO:0032303 | regulation of icosanoid secretion | Biological process | 2 | 17 | 169 | 17653 | 12.2888966237383 | 0.0112751312171173 | 0.545600938719608 | 1.94787839521157 | 0.0118343195266272 | CYP4F2//MIF// |
| GO:0032891 | negative regulation of organic acid transport | Biological process | 2 | 17 | 169 | 17653 | 12.2888966237383 | 0.0112751312171173 | 0.545600938719608 | 1.94787839521157 | 0.0118343195266272 | CYP4F2//OSR1// |
| GO:0071380 | cellular response to prostaglandin E stimulus | Biological process | 2 | 17 | 169 | 17653 | 12.2888966237383 | 0.0112751312171173 | 0.545600938719608 | 1.94787839521157 | 0.0118343195266272 | PRKAA1//SFRP1// |
| GO:0090185 | negative regulation of kidney development | Biological process | 2 | 17 | 169 | 17653 | 12.2888966237383 | 0.0112751312171173 | 0.545600938719608 | 1.94787839521157 | 0.0118343195266272 | OSR1//PAX8// |
| GO:0044085 | cellular component biogenesis | Biological process | 43 | 3209 | 169 | 17653 | 1.39968579494432 | 0.0112807123304149 | 0.545600938719608 | 1.9476634755831 | 0.254437869822485 | UBXN2B//TP53INP2//THUMPD1//SHQ1//RHOF//TWF1//ASF1B//HAT1//RPP14//CDH7//BDNF//BSN//MCIDAS//FAM161A//DYNLL1//BRK1//ISCU//ARFIP2//ARHGEF6//BAHD1//GPC1//EP300//TRABD2B//FERMT3//ABCA1//VPS37D//KCTD18//P2RX2//SPTBN5//FGFRL1//SRF//ARHGAP35//PRKAA1//CNIH2//GOSR2//DPYS//GRIN2B//YWHAB//SFRP1//MIF//KIF2A//KMT2D//NUDT16// |
| GO:0045893 | positive regulation of transcription, DNA-templated | Biological process | 23 | 1450 | 169 | 17653 | 1.65688226892471 | 0.0113086935306837 | 0.545600938719608 | 1.94658756529456 | 0.136094674556213 | SRF//EP300//HEYL//MICAL2//OSR1//FOXO1//ATP1B4//APBB1//MCIDAS//LMO2//FOXP3//ARID4B//PAX8//KMT2D//CREB3L3//OGT//PRKAA1//NPNT//NDP//ATXN7L3//TP53INP2//GPATCH3//SFRP1// |
| GO:1903508 | positive regulation of nucleic acid-templated transcription | Biological process | 23 | 1450 | 169 | 17653 | 1.65688226892471 | 0.0113086935306837 | 0.545600938719608 | 1.94658756529456 | 0.136094674556213 | SRF//EP300//HEYL//MICAL2//FOXO1//APBB1//NDP//FOXP3//ATXN7L3//TP53INP2//GPATCH3//SFRP1//PAX8//OSR1//ATP1B4//MCIDAS//LMO2//ARID4B//KMT2D//CREB3L3//OGT//PRKAA1//NPNT// |
| GO:0120034 | positive regulation of plasma membrane bounded cell projection assembly | Biological process | 4 | 91 | 169 | 17653 | 4.59145588139671 | 0.0113673481924697 | 0.545600938719608 | 1.94434083695685 | 0.0236686390532544 | BRK1//ARHGAP35//SRF//DYNLL1// |
| GO:1902680 | positive regulation of RNA biosynthetic process | Biological process | 23 | 1451 | 169 | 17653 | 1.65574037900815 | 0.0113959026596752 | 0.545600938719608 | 1.94325126902772 | 0.136094674556213 | SRF//EP300//HEYL//MICAL2//FOXO1//APBB1//NDP//FOXP3//ATXN7L3//TP53INP2//GPATCH3//SFRP1//PAX8//OSR1//ATP1B4//MCIDAS//LMO2//ARID4B//KMT2D//CREB3L3//OGT//PRKAA1//NPNT// |
| GO:0061383 | trabecula morphogenesis | Biological process | 3 | 49 | 169 | 17653 | 6.39524212051685 | 0.0115054832873447 | 0.545600938719608 | 1.93909513409062 | 0.0177514792899408 | UBE4B//SFRP1//SRF// |
| GO:2000649 | regulation of sodium ion transmembrane transporter activity | Biological process | 3 | 49 | 169 | 17653 | 6.39524212051685 | 0.0115054832873447 | 0.545600938719608 | 1.93909513409062 | 0.0177514792899408 | OSR1//GPD1L//SCN3B// |
| GO:0010975 | regulation of neuron projection development | Biological process | 10 | 450 | 169 | 17653 | 2.32123602892834 | 0.0115370645917062 | 0.545600938719608 | 1.93790467591078 | 0.0591715976331361 | ARHGAP35//APBB1//TWF1//CNTN2//SRF//BDNF//SEMA4B//SEMA4G//ZSWIM6//SFRP1// |
| GO:0016570 | histone modification | Biological process | 10 | 450 | 169 | 17653 | 2.32123602892834 | 0.0115370645917062 | 0.545600938719608 | 1.93790467591078 | 0.0591715976331361 | ATXN7L3//EP300//ARID4B//HDAC11//FOXP3//PRKAA1//APBB1//HAT1//OGT//KMT2D// |
| GO:0022008 | neurogenesis | Biological process | 24 | 1536 | 169 | 17653 | 1.63211908284024 | 0.0115403215978846 | 0.545600938719608 | 1.93778208838648 | 0.142011834319527 | SRF//APBB1//EFNA3//GPC1//ARHGAP35//NCAM1//SPTBN5//PRKCQ//BDNF//CNTN2//UNC5A//SFRP1//TWF1//HDAC11//BTG2//UBE4B//GNAO1//VAPA//HEYL//CAMK2A//SEMA4B//SEMA4G//NPTX1//ZSWIM6// |
| GO:0072080 | nephron tubule development | Biological process | 4 | 92 | 169 | 17653 | 4.54154875225109 | 0.0117974249337058 | 0.545600938719608 | 1.92821277736548 | 0.0236686390532544 | NPNT//PAX8//HEYL//OSR1// |
| GO:0010033 | response to organic substance | Biological process | 43 | 3218 | 169 | 17653 | 1.39577119825243 | 0.0118268416171026 | 0.545600938719608 | 1.92713121914225 | 0.254437869822485 | FAF2//EP300//CHRM1//FOXO1//FGFRL1//POLR2E//SRF//P2RX2//SFRP1//BTG2//IL4R//SH3BP4//UBE4B//TOB1//NPNT//FAM89B//CTDSPL2//CREB3L3//PRKAA1//MIF//CDKN2D//OGT//KMT2D//PSMB8//GNAO1//DYNLL1//GOSR2//CAMK2A//BDNF//GPC1//SMPD1//GRIN2B//PRKCQ//NCAM1//HEYL//ABCA1//OSR1//PTPN18//PAX8//SP5//CASP7//LMO2//SRSF3// |
| GO:0032890 | regulation of organic acid transport | Biological process | 3 | 50 | 169 | 17653 | 6.26733727810651 | 0.0121553154115938 | 0.545600938719608 | 1.9152337674058 | 0.0177514792899408 | CYP4F2//MIF//OSR1// |
| GO:0046635 | positive regulation of alpha-beta T cell activation | Biological process | 3 | 50 | 169 | 17653 | 6.26733727810651 | 0.0121553154115938 | 0.545600938719608 | 1.9152337674058 | 0.0177514792899408 | FOXP3//IL4R//PRKCQ// |
| GO:0030219 | megakaryocyte differentiation | Biological process | 4 | 93 | 169 | 17653 | 4.49271489470001 | 0.0122378215090652 | 0.545600938719608 | 1.91229588536011 | 0.0236686390532544 | EP300//SRF//PRKCQ//KMT2D// |
| GO:0044267 | cellular protein metabolic process | Biological process | 66 | 5421 | 169 | 17653 | 1.27173418297679 | 0.0124884912515117 | 0.545600938719608 | 1.90349002605514 | 0.390532544378698 | CDKN2D//GRIN2B//NCAM1//SPTBN5//PSMB8//YWHAB//CAMK2A//PRKAA1//UBE4B//MGRN1//COPS7B//ABHD17B//TULP4//CHRM1//STK35//PTPRT//PTPN18//CTDSPL2//LHPP//EP300//FOXO1//HAT1//BTG2//OGT//ABCA1//SPRN//PYURF//TP53INP2//ARHGEF6//CASP7//ATXN7L3//CRIM1//CNTN2//B4GALT5//ARID4B//HDAC11//USP54//NUP214//TOB1//PURA//MEX3A//PRKCQ//OSR1//RAB3B//ZDHHC22//ST6GALNAC6//FAF2//FOXP3//MIF//PPP1R26//GPD1L//SMPD1//UBXN2B//ARMC8//SFRP1//TWF1//ENAM//NUCB1//APBB1//KMT2D//POLR2E//SH3BP5//NPNT//FAM161A//EFNA3//TRABD2B// |
| GO:0003012 | muscle system process | Biological process | 10 | 456 | 169 | 17653 | 2.29069344960033 | 0.0125653800610557 | 0.545600938719608 | 1.90082437090563 | 0.0591715976331361 | CHRM1//GNAO1//MYL10//NPNT//SRF//TWF1//P2RX2//SCN3B//GPD1L//FOXO1// |
| GO:0006677 | glycosylceramide metabolic process | Biological process | 2 | 18 | 169 | 17653 | 11.6061801446417 | 0.0126054262686675 | 0.545600938719608 | 1.89944246351392 | 0.0118343195266272 | PRKAA1//ST6GALNAC6// |
| GO:0007221 | positive regulation of transcription of Notch receptor target | Biological process | 2 | 18 | 169 | 17653 | 11.6061801446417 | 0.0126054262686675 | 0.545600938719608 | 1.89944246351392 | 0.0118343195266272 | EP300//HEYL// |
| GO:0030952 | establishment or maintenance of cytoskeleton polarity | Biological process | 2 | 18 | 169 | 17653 | 11.6061801446417 | 0.0126054262686675 | 0.545600938719608 | 1.89944246351392 | 0.0118343195266272 | ARHGAP35//RHOF// |
| GO:0045076 | regulation of interleukin-2 biosynthetic process | Biological process | 2 | 18 | 169 | 17653 | 11.6061801446417 | 0.0126054262686675 | 0.545600938719608 | 1.89944246351392 | 0.0118343195266272 | FOXP3//PRKCQ// |
| GO:0046639 | negative regulation of alpha-beta T cell differentiation | Biological process | 2 | 18 | 169 | 17653 | 11.6061801446417 | 0.0126054262686675 | 0.545600938719608 | 1.89944246351392 | 0.0118343195266272 | IL4R//FOXP3// |
| GO:0097062 | dendritic spine maintenance | Biological process | 2 | 18 | 169 | 17653 | 11.6061801446417 | 0.0126054262686675 | 0.545600938719608 | 1.89944246351392 | 0.0118343195266272 | ABHD17B//GRIN2B// |
| GO:2000696 | regulation of epithelial cell differentiation involved in kidney development | Biological process | 2 | 18 | 169 | 17653 | 11.6061801446417 | 0.0126054262686675 | 0.545600938719608 | 1.89944246351392 | 0.0118343195266272 | PAX8//OSR1// |
| GO:0061326 | renal tubule development | Biological process | 4 | 94 | 169 | 17653 | 4.44492005539469 | 0.0126886219101277 | 0.545600938719608 | 1.89658554333688 | 0.0236686390532544 | NPNT//PAX8//HEYL//OSR1// |
| GO:0030514 | negative regulation of BMP signaling pathway | Biological process | 3 | 51 | 169 | 17653 | 6.14444831186913 | 0.0128258404848846 | 0.545600938719608 | 1.89191416571031 | 0.0177514792899408 | TOB1//CTDSPL2//SFRP1// |
| GO:0046467 | membrane lipid biosynthetic process | Biological process | 5 | 145 | 169 | 17653 | 3.60191797592328 | 0.0129051777414091 | 0.545600938719608 | 1.889236009595 | 0.029585798816568 | ST6GALNAC6//PYURF//VAPA//PRKAA1//SMPD1// |
| GO:0045664 | regulation of neuron differentiation | Biological process | 12 | 600 | 169 | 17653 | 2.0891124260355 | 0.0129079033262069 | 0.545600938719608 | 1.8891442959062 | 0.0710059171597633 | SFRP1//ARHGAP35//APBB1//TWF1//CNTN2//HEYL//SRF//BDNF//SEMA4B//SEMA4G//ZSWIM6//EFNA3// |
| GO:0042221 | response to chemical | Biological process | 58 | 4655 | 169 | 17653 | 1.30148787014027 | 0.0131690718035607 | 0.550733003250073 | 1.88044483436226 | 0.343195266272189 | P2RX2//FAF2//RALBP1//EP300//CHRM1//EFNA3//GPC1//ARHGAP35//NCAM1//SPTBN5//PRKCQ//BDNF//CNTN2//UNC5A//ABCA1//OGT//HAT1//FOXO1//FGFRL1//POLR2E//SFRP1//SRF//APBB1//MIF//BTG2//IL4R//SH3BP4//UBE4B//TOB1//NPNT//FAM89B//CTDSPL2//CREB3L3//PRKAA1//CDKN2D//KMT2D//PSMB8//GNAO1//GPX8//DYNLL1//GOSR2//CAMK2A//SMPD1//GRIN2B//SEMA4B//SEMA4G//RAB3B//HEYL//OSR1//PTPN18//PAX8//SP5//NUCB1//CASP7//LMO2//DDAH1//ZSWIM6//SRSF3// |
| GO:0051253 | negative regulation of RNA metabolic process | Biological process | 21 | 1306 | 169 | 17653 | 1.67960799949256 | 0.0133405549575837 | 0.550733003250073 | 1.87482610370259 | 0.124260355029586 | OSR1//EP300//FOXO1//HEYL//ARHGAP35//KCNIP3//APBB1//SP5//FOXP3//BTG2//BAHD1//KMT2D//HAT1//PURA//SFRP1//YWHAB//BTAF1//TOB1//SRF//NUDT16//SHQ1// |
| GO:0016311 | dephosphorylation | Biological process | 10 | 461 | 169 | 17653 | 2.26584861825976 | 0.0134740042709839 | 0.550733003250073 | 1.87050331932085 | 0.0591715976331361 | PTPRT//PTPN18//CTDSPL2//LHPP//NPNT//PPP1R26//SMPD1//YWHAB//UBXN2B//NUDT16// |
| GO:0030042 | actin filament depolymerization | Biological process | 3 | 52 | 169 | 17653 | 6.02628584433318 | 0.0135171522723586 | 0.550733003250073 | 1.86911479379882 | 0.0177514792899408 | TWF1//SPTBN5//MICAL2// |
| GO:0008154 | actin polymerization or depolymerization | Biological process | 6 | 203 | 169 | 17653 | 3.0873582650771 | 0.0135568807649927 | 0.550733003250073 | 1.8678402236241 | 0.0355029585798817 | TWF1//MICAL2//ARHGAP35//BRK1//ARFIP2//SPTBN5// |
| GO:0016573 | histone acetylation | Biological process | 5 | 147 | 169 | 17653 | 3.55291228917602 | 0.0136260122014366 | 0.550733003250073 | 1.86563122650669 | 0.029585798816568 | FOXP3//EP300//APBB1//HAT1//OGT// |
| GO:0030220 | platelet formation | Biological process | 2 | 19 | 169 | 17653 | 10.9953285580816 | 0.0140006352991337 | 0.550733003250073 | 1.85385225713261 | 0.0118343195266272 | EP300//SRF// |
| GO:0042789 | mRNA transcription by RNA polymerase II | Biological process | 2 | 19 | 169 | 17653 | 10.9953285580816 | 0.0140006352991337 | 0.550733003250073 | 1.85385225713261 | 0.0118343195266272 | LMO2//SRF// |
| GO:0072079 | nephron tubule formation | Biological process | 2 | 19 | 169 | 17653 | 10.9953285580816 | 0.0140006352991337 | 0.550733003250073 | 1.85385225713261 | 0.0118343195266272 | OSR1//PAX8// |
| GO:0016569 | covalent chromatin modification | Biological process | 10 | 464 | 169 | 17653 | 2.25119873495205 | 0.0140424159332303 | 0.550733003250073 | 1.85255816740507 | 0.0591715976331361 | ATXN7L3//EP300//ARID4B//HDAC11//FOXP3//PRKAA1//APBB1//HAT1//OGT//KMT2D// |
| GO:0001657 | ureteric bud development | Biological process | 4 | 97 | 169 | 17653 | 4.30744830110413 | 0.0141042553460507 | 0.550733003250073 | 1.85064983808984 | 0.0236686390532544 | NPNT//PAX8//OSR1//SFRP1// |
| GO:0009952 | anterior/posterior pattern specification | Biological process | 6 | 205 | 169 | 17653 | 3.05723769663732 | 0.0141684971500672 | 0.550733003250073 | 1.84867621284686 | 0.0355029585798817 | EP300//OSR1//SRF//SFRP1//HOXC9//BTG2// |
| GO:0060537 | muscle tissue development | Biological process | 9 | 396 | 169 | 17653 | 2.37399139322216 | 0.0141930761858154 | 0.550733003250073 | 1.84792346601283 | 0.0532544378698225 | UBE4B//EP300//HEYL//BTG2//PRKAA1//P2RX2//SRF//OSR1//GPC1// |
| GO:0006476 | protein deacetylation | Biological process | 4 | 98 | 169 | 17653 | 4.26349474701123 | 0.0145974700753433 | 0.550733003250073 | 1.83572240637478 | 0.0236686390532544 | ARID4B//HDAC11//FOXP3//EP300// |
| GO:0072163 | mesonephric epithelium development | Biological process | 4 | 98 | 169 | 17653 | 4.26349474701123 | 0.0145974700753433 | 0.550733003250073 | 1.83572240637478 | 0.0236686390532544 | OSR1//NPNT//SFRP1//PAX8// |
| GO:0072164 | mesonephric tubule development | Biological process | 4 | 98 | 169 | 17653 | 4.26349474701123 | 0.0145974700753433 | 0.550733003250073 | 1.83572240637478 | 0.0236686390532544 | OSR1//NPNT//SFRP1//PAX8// |
| GO:0007517 | muscle organ development | Biological process | 9 | 398 | 169 | 17653 | 2.3620617882311 | 0.0146223930531057 | 0.550733003250073 | 1.8349815463446 | 0.0532544378698225 | UBE4B//EP300//HEYL//BTG2//PRKAA1//P2RX2//FGFRL1//GPC1//EVC// |
| GO:0048863 | stem cell differentiation | Biological process | 7 | 268 | 169 | 17653 | 2.72831846683741 | 0.0148263857314084 | 0.550733003250073 | 1.82896470522311 | 0.0414201183431953 | SEMA4B//SEMA4G//SRF//SFRP1//LMO2//PSMB8//OSR1// |
| GO:0050922 | negative regulation of chemotaxis | Biological process | 3 | 54 | 169 | 17653 | 5.80309007232084 | 0.0149624674410161 | 0.550733003250073 | 1.8249967816283 | 0.0177514792899408 | MIF//SEMA4B//SEMA4G// |
| GO:0007413 | axonal fasciculation | Biological process | 2 | 20 | 169 | 17653 | 10.4455621301775 | 0.0154594087751457 | 0.550733003250073 | 1.81080711912413 | 0.0118343195266272 | ARHGAP35//CNTN2// |
| GO:0036344 | platelet morphogenesis | Biological process | 2 | 20 | 169 | 17653 | 10.4455621301775 | 0.0154594087751457 | 0.550733003250073 | 1.81080711912413 | 0.0118343195266272 | EP300//SRF// |
| GO:0045724 | positive regulation of cilium assembly | Biological process | 2 | 20 | 169 | 17653 | 10.4455621301775 | 0.0154594087751457 | 0.550733003250073 | 1.81080711912413 | 0.0118343195266272 | DYNLL1//ARHGAP35// |
| GO:0048843 | negative regulation of axon extension involved in axon guidance | Biological process | 2 | 20 | 169 | 17653 | 10.4455621301775 | 0.0154594087751457 | 0.550733003250073 | 1.81080711912413 | 0.0118343195266272 | SEMA4B//SEMA4G// |
| GO:0072087 | renal vesicle development | Biological process | 2 | 20 | 169 | 17653 | 10.4455621301775 | 0.0154594087751457 | 0.550733003250073 | 1.81080711912413 | 0.0118343195266272 | PAX8//OSR1// |
| GO:0072234 | metanephric nephron tubule development | Biological process | 2 | 20 | 169 | 17653 | 10.4455621301775 | 0.0154594087751457 | 0.550733003250073 | 1.81080711912413 | 0.0118343195266272 | PAX8//OSR1// |
| GO:0106030 | neuron projection fasciculation | Biological process | 2 | 20 | 169 | 17653 | 10.4455621301775 | 0.0154594087751457 | 0.550733003250073 | 1.81080711912413 | 0.0118343195266272 | ARHGAP35//CNTN2// |
| GO:2000515 | negative regulation of CD4-positive, alpha-beta T cell activation | Biological process | 2 | 20 | 169 | 17653 | 10.4455621301775 | 0.0154594087751457 | 0.550733003250073 | 1.81080711912413 | 0.0118343195266272 | IL4R//FOXP3// |
| GO:0018393 | internal peptidyl-lysine acetylation | Biological process | 5 | 152 | 169 | 17653 | 3.4360401744005 | 0.0155457267228294 | 0.550733003250073 | 1.80838897099374 | 0.029585798816568 | EP300//FOXP3//APBB1//HAT1//OGT// |
| GO:0090288 | negative regulation of cellular response to growth factor stimulus | Biological process | 5 | 152 | 169 | 17653 | 3.4360401744005 | 0.0155457267228294 | 0.550733003250073 | 1.80838897099374 | 0.029585798816568 | FAM89B//TOB1//CTDSPL2//SFRP1//GPC1// |
| GO:0061387 | regulation of extent of cell growth | Biological process | 4 | 100 | 169 | 17653 | 4.17822485207101 | 0.0156163504157231 | 0.550733003250073 | 1.80642045442204 | 0.0236686390532544 | SRF//BDNF//SEMA4B//SEMA4G// |
| GO:0032370 | positive regulation of lipid transport | Biological process | 3 | 55 | 169 | 17653 | 5.69757934373319 | 0.0157166153522496 | 0.550733003250073 | 1.80364097560631 | 0.0177514792899408 | ABCA1//CYP4F2//MIF// |
| GO:0046513 | ceramide biosynthetic process | Biological process | 3 | 55 | 169 | 17653 | 5.69757934373319 | 0.0157166153522496 | 0.550733003250073 | 1.80364097560631 | 0.0177514792899408 | ST6GALNAC6//PRKAA1//SMPD1// |
| GO:0046928 | regulation of neurotransmitter secretion | Biological process | 3 | 55 | 169 | 17653 | 5.69757934373319 | 0.0157166153522496 | 0.550733003250073 | 1.80364097560631 | 0.0177514792899408 | RIMS4//CPLX1//CAMK2A// |
| GO:0045184 | establishment of protein localization | Biological process | 29 | 2012 | 169 | 17653 | 1.50557307045302 | 0.0159375422708427 | 0.555595867926336 | 1.79757865034938 | 0.171597633136095 | SPRN//NUP214//SRSF3//YWHAB//ZDHHC22//GOSR2//TOMM40//ARFIP2//RABL3//KCNIP3//RAB3B//MLPH//VPS37D//VPS53//CPLX1//FAF2//DYNLL1//SFRP1//CTDSPL2//ABCA1//FOXP3//APBB1//MIF//CNTN2//IL4R//CHRM1//NUCB1//PRKAA1//NPNT// |
| GO:0030148 | sphingolipid biosynthetic process | Biological process | 4 | 101 | 169 | 17653 | 4.13685628917921 | 0.0161421573096077 | 0.559843127614753 | 1.79203842469144 | 0.0236686390532544 | ST6GALNAC6//PRKAA1//SMPD1//VAPA// |
| GO:0001823 | mesonephros development | Biological process | 4 | 102 | 169 | 17653 | 4.09629887457942 | 0.0166789660986876 | 0.575509427170532 | 1.77783087405508 | 0.0236686390532544 | OSR1//NPNT//SFRP1//PAX8// |
| GO:0010769 | regulation of cell morphogenesis involved in differentiation | Biological process | 7 | 275 | 169 | 17653 | 2.65887036040882 | 0.0168459883127176 | 0.577078184519794 | 1.77350350494592 | 0.0414201183431953 | CNTN2//SRF//BDNF//SEMA4B//SEMA4G//ARHGAP35//ZSWIM6// |
| GO:0045595 | regulation of cell differentiation | Biological process | 26 | 1761 | 169 | 17653 | 1.54221814528458 | 0.0169531395087727 | 0.577078184519794 | 1.77074986412289 | 0.153846153846154 | SFRP1//ABCA1//BDNF//ARHGAP35//APBB1//TWF1//OSR1//CNTN2//FOXP3//SRF//FOXO1//IL4R//EP300//PRKCQ//KMT2D//EFNA3//HEYL//TOB1//NPNT//SEMA4B//SEMA4G//PAX8//LMO2//PSMB8//ZSWIM6//GPC1// |
| GO:0042094 | interleukin-2 biosynthetic process | Biological process | 2 | 21 | 169 | 17653 | 9.94815440969287 | 0.0169804167853673 | 0.577078184519794 | 1.77005165417665 | 0.0118343195266272 | FOXP3//PRKCQ// |
| GO:0051261 | protein depolymerization | Biological process | 4 | 103 | 169 | 17653 | 4.05652898259321 | 0.017226842259825 | 0.581691741959354 | 1.76379432294755 | 0.0236686390532544 | TWF1//MICAL2//KIF2A//SPTBN5// |
| GO:0097306 | cellular response to alcohol | Biological process | 3 | 57 | 169 | 17653 | 5.4976642790408 | 0.0172881916507216 | 0.581691741959354 | 1.76225043166452 | 0.0177514792899408 | PRKAA1//SFRP1//ABCA1// |
| GO:0065007 | biological regulation | Biological process | 131 | 12351 | 169 | 17653 | 1.10790109226237 | 0.0174514114653451 | 0.584276711584797 | 1.75816944166636 | 0.775147928994083 | CDKN2D//OSR1//EP300//FOXO1//HEYL//ARHGAP35//KCNIP3//APBB1//SP5//FOXP3//BTG2//GRIN2B//NCAM1//SPTBN5//PSMB8//YWHAB//CAMK2A//PRKAA1//CRIM1//PRKCQ//TP53INP2//SFRP1//GPD1L//IL4R//CREB3L3//MIF//P2RX2//CHRM1//DDAH1//CYP4F2//SRF//NUP214//TWF1//AKAP10//TRABD2B//NDP//GNAO1//RALBP1//PTPRT//FGFRL1//SMPD1//OGT//VAPA//SH3BP5//BDNF//CARHSP1//MCIDAS//BAHD1//KMT2D//HAT1//ARID4B//ZNF618//ATP1B4//MYCBP//PFDN1//ASF1B//ZNF395//HDAC11//DYNLL1//HOXC9//PURA//NUDT16//ISCU//CLEC1A//ABCA1//GNAZ//GPR153//ARHGEF6//GPR3//EVC//ADAM11//FERMT3//ARFIP2//RHOF//CPLX1//TTC28//BRK1//TOB1//SH3BP4//POLR2E//UBE4B//CASP7//NPNT//PYURF//SCN3B//CSPG5//PAX8//MICAL2//PPP1R26//CNTN2//MGRN1//EFNA3//ATXN7L3//GPATCH3//BTAF1//MEX3A//RAB3B//DPYS//ENAM//KIF2A//SEMA4B//FAM89B//SEMA4G//CTDSPL2//POLR3H//RABL3//GOSR2//UNC5A//PTPN18//GPC1//SLC26A1//RIMS4//CNIH2//SHQ1//FAF2//UBXN2B//LMO2//SRSF3//CGNL1//BEST1//ANO3//DENND6A//ST5//ZNF385B//BSN//LYPD6//FAM161A//ABHD17B//ZSWIM6//NUCB1//SESTD1// |
| GO:0048523 | negative regulation of cellular process | Biological process | 59 | 4821 | 169 | 17653 | 1.27834093690204 | 0.0177652923522163 | 0.586622265800254 | 1.75042764120535 | 0.349112426035503 | OSR1//EP300//FOXO1//HEYL//ARHGAP35//KCNIP3//APBB1//SP5//FOXP3//BTG2//MIF//TWF1//BAHD1//KMT2D//HAT1//CDKN2D//PRKAA1//SFRP1//TOB1//DDAH1//SH3BP4//FGFRL1//SRF//PAX8//ABCA1//BDNF//PPP1R26//CRIM1//PSMB8//MGRN1//YWHAB//PURA//BTAF1//MEX3A//SMPD1//TRABD2B//FAM89B//CTDSPL2//OGT//GPD1L//GPATCH3//GPC1//DYNLL1//IL4R//CNTN2//PRKCQ//SEMA4B//SEMA4G//CGNL1//SPTBN5//POLR2E//NUP214//SH3BP5//PTPN18//GRIN2B//CNIH2//NUDT16//SHQ1//LYPD6// |
| GO:0003205 | cardiac chamber development | Biological process | 5 | 158 | 169 | 17653 | 3.30555763613213 | 0.0180781849486892 | 0.586622265800254 | 1.74284517487494 | 0.029585798816568 | HEYL//UBE4B//PAX8//SRF//FGFRL1// |
| GO:0006475 | internal protein amino acid acetylation | Biological process | 5 | 158 | 169 | 17653 | 3.30555763613213 | 0.0180781849486892 | 0.586622265800254 | 1.74284517487494 | 0.029585798816568 | EP300//FOXP3//APBB1//HAT1//OGT// |
| GO:0010508 | positive regulation of autophagy | Biological process | 4 | 105 | 169 | 17653 | 3.97926176387715 | 0.0183560482784019 | 0.586622265800254 | 1.73622080873419 | 0.0236686390532544 | FOXO1//SH3BP4//PRKAA1//TP53INP2// |
| GO:0006497 | protein lipidation | Biological process | 5 | 159 | 169 | 17653 | 3.28476796546463 | 0.0185251782164885 | 0.586622265800254 | 1.73223760531542 | 0.029585798816568 | SPRN//PYURF//ZDHHC22//ABHD17B//ABCA1// |
| GO:0018394 | peptidyl-lysine acetylation | Biological process | 5 | 159 | 169 | 17653 | 3.28476796546463 | 0.0185251782164885 | 0.586622265800254 | 1.73223760531542 | 0.029585798816568 | EP300//FOXP3//APBB1//HAT1//OGT// |
| GO:0120032 | regulation of plasma membrane bounded cell projection assembly | Biological process | 5 | 159 | 169 | 17653 | 3.28476796546463 | 0.0185251782164885 | 0.586622265800254 | 1.73223760531542 | 0.029585798816568 | TWF1//BRK1//ARHGAP35//SRF//DYNLL1// |
| GO:0032753 | positive regulation of interleukin-4 production | Biological process | 2 | 22 | 169 | 17653 | 9.49596557288865 | 0.0185623487921417 | 0.586622265800254 | 1.73136707106294 | 0.0118343195266272 | FOXP3//PRKCQ// |
| GO:0048745 | smooth muscle tissue development | Biological process | 2 | 22 | 169 | 17653 | 9.49596557288865 | 0.0185623487921417 | 0.586622265800254 | 1.73136707106294 | 0.0118343195266272 | SRF//OSR1// |
| GO:0071379 | cellular response to prostaglandin stimulus | Biological process | 2 | 22 | 169 | 17653 | 9.49596557288865 | 0.0185623487921417 | 0.586622265800254 | 1.73136707106294 | 0.0118343195266272 | PRKAA1//SFRP1// |
| GO:0072538 | T-helper 17 type immune response | Biological process | 2 | 22 | 169 | 17653 | 9.49596557288865 | 0.0185623487921417 | 0.586622265800254 | 1.73136707106294 | 0.0118343195266272 | PRKCQ//FOXP3// |
| GO:1902668 | negative regulation of axon guidance | Biological process | 2 | 22 | 169 | 17653 | 9.49596557288865 | 0.0185623487921417 | 0.586622265800254 | 1.73136707106294 | 0.0118343195266272 | SEMA4B//SEMA4G// |
| GO:0015833 | peptide transport | Biological process | 28 | 1951 | 169 | 17653 | 1.49910681519718 | 0.0187812169025796 | 0.587712443892776 | 1.72627627165705 | 0.165680473372781 | SPRN//NUP214//SRSF3//YWHAB//ZDHHC22//GOSR2//TOMM40//ARFIP2//RABL3//KCNIP3//RAB3B//MLPH//VPS37D//VPS53//CPLX1//FAF2//DYNLL1//SFRP1//CTDSPL2//ABCA1//FOXP3//APBB1//MIF//IL4R//CHRM1//NUCB1//PRKAA1//PAX8// |
| GO:0009605 | response to external stimulus | Biological process | 32 | 2306 | 169 | 17653 | 1.44951425917468 | 0.0188828761956188 | 0.587712443892776 | 1.72393185427788 | 0.189349112426035 | CREB3L3//FOXP3//RALBP1//BTG2//EFNA3//GPC1//ARHGAP35//NCAM1//SPTBN5//PRKCQ//BDNF//CNTN2//UNC5A//ABCA1//OGT//HAT1//FOXO1//SFRP1//MIF//ANO3//PRKAA1//P2RX2//CDKN2D//GPATCH3//IL4R//SRF//SEMA4B//SEMA4G//BEST1//POLR3H//NPNT//ZSWIM6// |
| GO:0006939 | smooth muscle contraction | Biological process | 4 | 106 | 169 | 17653 | 3.94172155855755 | 0.0189374987667422 | 0.587712443892776 | 1.72267738243597 | 0.0236686390532544 | CHRM1//NPNT//SRF//P2RX2// |
| GO:1902305 | regulation of sodium ion transmembrane transport | Biological process | 3 | 59 | 169 | 17653 | 5.31130277805636 | 0.0189444496183092 | 0.587712443892776 | 1.72251800750186 | 0.0177514792899408 | GPD1L//SCN3B//OSR1// |
| GO:0007169 | transmembrane receptor protein tyrosine kinase signaling pathway | Biological process | 13 | 711 | 169 | 17653 | 1.90987774532078 | 0.0193468063820538 | 0.591222302273517 | 1.71339071463061 | 0.0769230769230769 | FOXO1//FGFRL1//POLR2E//BDNF//PTPN18//GPC1//OGT//PRKCQ//CRIM1//BRK1//EFNA3//GRIN2B//PTPRT// |
| GO:0060491 | regulation of cell projection assembly | Biological process | 5 | 161 | 169 | 17653 | 3.24396339446507 | 0.0194409175334368 | 0.591222302273517 | 1.71128324196607 | 0.029585798816568 | TWF1//BRK1//ARHGAP35//SRF//DYNLL1// |
| GO:0050789 | regulation of biological process | Biological process | 125 | 11709 | 169 | 17653 | 1.11512107461969 | 0.019647934076615 | 0.591222302273517 | 1.7066831076954 | 0.739644970414201 | CDKN2D//OSR1//EP300//FOXO1//HEYL//ARHGAP35//KCNIP3//APBB1//SP5//FOXP3//BTG2//GRIN2B//NCAM1//SPTBN5//PSMB8//YWHAB//CAMK2A//PRKAA1//CRIM1//PRKCQ//SFRP1//GPD1L//IL4R//CREB3L3//MIF//CHRM1//SRF//NUP214//TWF1//TRABD2B//NDP//GNAO1//RALBP1//PTPRT//AKAP10//FGFRL1//SMPD1//OGT//VAPA//SH3BP5//BDNF//CARHSP1//MCIDAS//BAHD1//KMT2D//HAT1//ARID4B//ZNF618//ATP1B4//MYCBP//PFDN1//ASF1B//ZNF395//HDAC11//DYNLL1//HOXC9//PURA//NUDT16//CLEC1A//ABCA1//GNAZ//GPR153//ARHGEF6//GPR3//EVC//ADAM11//FERMT3//DDAH1//ARFIP2//RHOF//TTC28//BRK1//TOB1//SH3BP4//POLR2E//UBE4B//CASP7//NPNT//PYURF//SCN3B//CSPG5//TP53INP2//P2RX2//PAX8//MICAL2//PPP1R26//CNTN2//MGRN1//EFNA3//ATXN7L3//GPATCH3//BTAF1//MEX3A//CPLX1//RAB3B//ENAM//KIF2A//SEMA4B//FAM89B//SEMA4G//CTDSPL2//CYP4F2//POLR3H//RABL3//GOSR2//UNC5A//PTPN18//GPC1//SHQ1//UBXN2B//LMO2//SRSF3//RIMS4//CGNL1//BEST1//ZNF385B//BSN//LYPD6//FAM161A//ABHD17B//ZSWIM6//CNIH2//NUCB1//SESTD1//DENND6A// |
| GO:0006643 | membrane lipid metabolic process | Biological process | 6 | 221 | 169 | 17653 | 2.83589922086267 | 0.0197684861078586 | 0.591222302273517 | 1.70402658815611 | 0.0355029585798817 | ST6GALNAC6//PYURF//PLA2G15//SMPD1//VAPA//PRKAA1// |
| GO:0051252 | regulation of RNA metabolic process | Biological process | 50 | 3984 | 169 | 17653 | 1.31093902236164 | 0.020093361556772 | 0.591222302273517 | 1.69694740106006 | 0.29585798816568 | OSR1//EP300//FOXO1//HEYL//ARHGAP35//KCNIP3//APBB1//SP5//FOXP3//BTG2//SRF//BAHD1//KMT2D//HAT1//ZNF618//ATP1B4//MYCBP//PFDN1//ASF1B//PRKCQ//ZNF395//HDAC11//DYNLL1//CARHSP1//HOXC9//ARID4B//PURA//OGT//MICAL2//NDP//ATXN7L3//TP53INP2//GPATCH3//SFRP1//PAX8//YWHAB//BTAF1//TOB1//CTDSPL2//PSMB8//NUP214//MCIDAS//LMO2//CREB3L3//SRSF3//CAMK2A//PRKAA1//NUDT16//SHQ1//NPNT// |
| GO:0043279 | response to alkaloid | Biological process | 4 | 108 | 169 | 17653 | 3.86872671488056 | 0.020134380827174 | 0.591222302273517 | 1.69606172128259 | 0.0236686390532544 | PRKAA1//SMPD1//GNAO1//CASP7// |
| GO:0071453 | cellular response to oxygen levels | Biological process | 6 | 222 | 169 | 17653 | 2.82312490004798 | 0.0201619726456442 | 0.591222302273517 | 1.69546697881349 | 0.0355029585798817 | EP300//PSMB8//FOXO1//PRKAA1//SFRP1//DDAH1// |
| GO:0032660 | regulation of interleukin-17 production | Biological process | 2 | 23 | 169 | 17653 | 9.08309750450219 | 0.020203913386049 | 0.591222302273517 | 1.69456450197129 | 0.0118343195266272 | FOXP3//PRKCQ// |
| GO:0034505 | tooth mineralization | Biological process | 2 | 23 | 169 | 17653 | 9.08309750450219 | 0.020203913386049 | 0.591222302273517 | 1.69456450197129 | 0.0118343195266272 | FOXO1//ENAM// |
| GO:0044458 | motile cilium assembly | Biological process | 2 | 23 | 169 | 17653 | 9.08309750450219 | 0.020203913386049 | 0.591222302273517 | 1.69456450197129 | 0.0118343195266272 | MCIDAS//DYNLL1// |
| GO:0072170 | metanephric tubule development | Biological process | 2 | 23 | 169 | 17653 | 9.08309750450219 | 0.020203913386049 | 0.591222302273517 | 1.69456450197129 | 0.0118343195266272 | PAX8//OSR1// |
| GO:0072243 | metanephric nephron epithelium development | Biological process | 2 | 23 | 169 | 17653 | 9.08309750450219 | 0.020203913386049 | 0.591222302273517 | 1.69456450197129 | 0.0118343195266272 | PAX8//OSR1// |
| GO:2000191 | regulation of fatty acid transport | Biological process | 2 | 23 | 169 | 17653 | 9.08309750450219 | 0.020203913386049 | 0.591222302273517 | 1.69456450197129 | 0.0118343195266272 | CYP4F2//MIF// |
| GO:0006810 | transport | Biological process | 61 | 5048 | 169 | 17653 | 1.2622410656514 | 0.0202814688936058 | 0.591222302273517 | 1.69290059424572 | 0.36094674556213 | SPRN//NUP214//ISLR//FERMT3//SRSF3//YWHAB//ZDHHC22//GOSR2//TOMM40//SLC26A1//P2RX2//SCN3B//CAMK2A//BEST1//SLC39A2//RALBP1//ABCA1//PCTP//ANO3//ARFIP2//RABL3//KCNIP3//RAB3B//MLPH//CPLX1//CNIH2//SPTBN5//DYNLL1//VAPA//KIF2A//SH3BP4//VPS53//MGRN1//ATP1B4//GPD1L//VPS37D//FAF2//CNTN2//CYP4F2//BRK1//CHRM1//IL4R//ARMC8//MIF//RHOF//SFRP1//CTDSPL2//CSPG5//RIMS4//FOXP3//APBB1//MOGAT2//GNAO1//PSMB8//PRKAA1//GRIN2B//NDUFA4//NUCB1//SESTD1//OSR1//PAX8// |
| GO:0048731 | system development | Biological process | 58 | 4760 | 169 | 17653 | 1.27277857888718 | 0.020649940860713 | 0.597108561114827 | 1.68508118778011 | 0.343195266272189 | EVC//FGFRL1//FOXO1//SRF//OSR1//PAX8//NPNT//SFRP1//SEMA4B//SEMA4G//ARHGAP35//NDP//MICAL2//FOXP3//HEYL//UBE4B//EP300//CSPG5//CHRM1//KIF2A//CRIM1//SCN3B//PURA//BDNF//SMPD1//CAMK2A//APBB1//EFNA3//GPC1//NCAM1//SPTBN5//PRKCQ//CNTN2//UNC5A//BSN//NPTX1//GRIN2B//MEA1//TWF1//HDAC11//BTG2//DYNLL1//GNAO1//VAPA//ENAM//KRTAP1-1//TMIE//IL4R//KMT2D//DDAH1//PRKAA1//HOXC9//P2RX2//PSMB8//SP5//ARID4B//LMO2//ZSWIM6// |
| GO:0072009 | nephron epithelium development | Biological process | 4 | 109 | 169 | 17653 | 3.83323380923945 | 0.0207499206831441 | 0.597108561114827 | 1.68298355904532 | 0.0236686390532544 | NPNT//PAX8//HEYL//OSR1// |
| GO:0006665 | sphingolipid metabolic process | Biological process | 5 | 164 | 169 | 17653 | 3.18462260066388 | 0.0208695344274419 | 0.597108561114827 | 1.6804872393796 | 0.029585798816568 | ST6GALNAC6//PLA2G15//SMPD1//VAPA//PRKAA1// |
| GO:0042158 | lipoprotein biosynthetic process | Biological process | 5 | 164 | 169 | 17653 | 3.18462260066388 | 0.0208695344274419 | 0.597108561114827 | 1.6804872393796 | 0.029585798816568 | ABCA1//SPRN//PYURF//ZDHHC22//ABHD17B// |
| GO:0009991 | response to extracellular stimulus | Biological process | 10 | 495 | 169 | 17653 | 2.11021457175303 | 0.0210187066399347 | 0.597108561114827 | 1.67739401125834 | 0.0591715976331361 | ABCA1//OGT//HAT1//FOXO1//SFRP1//ARHGAP35//PRKAA1//P2RX2//CDKN2D//SRF// |
| GO:0051234 | establishment of localization | Biological process | 62 | 5155 | 169 | 17653 | 1.25630427171873 | 0.0210370473825651 | 0.597108561114827 | 1.67701521487204 | 0.366863905325444 | SPRN//NUP214//ISLR//FERMT3//SRSF3//YWHAB//ZDHHC22//GOSR2//TOMM40//SLC26A1//P2RX2//SCN3B//CAMK2A//BEST1//SLC39A2//RALBP1//ABCA1//PCTP//ANO3//ARFIP2//RABL3//KCNIP3//RAB3B//MLPH//CPLX1//CNIH2//SPTBN5//DYNLL1//VAPA//KIF2A//SH3BP4//VPS53//MGRN1//ATP1B4//GPD1L//VPS37D//FAF2//CNTN2//CYP4F2//BRK1//CHRM1//IL4R//ARMC8//MIF//RHOF//NPNT//SFRP1//CTDSPL2//CSPG5//RIMS4//FOXP3//APBB1//MOGAT2//GNAO1//PSMB8//PRKAA1//GRIN2B//NDUFA4//NUCB1//SESTD1//OSR1//PAX8// |
| GO:0035556 | intracellular signal transduction | Biological process | 38 | 2871 | 169 | 17653 | 1.38255437459681 | 0.0211092181918237 | 0.597108561114827 | 1.67552785107247 | 0.224852071005917 | GRIN2B//NCAM1//SPTBN5//PSMB8//YWHAB//CAMK2A//PRKAA1//EP300//BTG2//ABCA1//GPR3//ARHGEF6//DDAH1//RALBP1//ARFIP2//RHOF//MIF//BRK1//SH3BP4//RABL3//RAB3B//FOXO1//OGT//ARHGAP35//GPATCH3//VAPA//SMPD1//SFRP1//MGRN1//P2RX2//CGNL1//NPNT//ZNF385B//GPD1L//CDKN2D//CARHSP1//PRKCQ//SH3BP5// |
| GO:0006936 | muscle contraction | Biological process | 8 | 355 | 169 | 17653 | 2.35392949412451 | 0.0211897167924824 | 0.597108561114827 | 1.67387484773867 | 0.0473372781065089 | CHRM1//NPNT//SRF//P2RX2//SCN3B//GPD1L//GNAO1//MYL10// |
| GO:0042886 | amide transport | Biological process | 28 | 1972 | 169 | 17653 | 1.48314269596841 | 0.0213335859392411 | 0.598668222850986 | 1.67093613832785 | 0.165680473372781 | SPRN//NUP214//SRSF3//YWHAB//ZDHHC22//GOSR2//TOMM40//ARFIP2//RABL3//KCNIP3//RAB3B//MLPH//VPS37D//VPS53//CPLX1//FAF2//DYNLL1//SFRP1//CTDSPL2//ABCA1//FOXP3//APBB1//MIF//IL4R//CHRM1//NUCB1//PRKAA1//PAX8// |
| GO:1903311 | regulation of mRNA metabolic process | Biological process | 7 | 289 | 169 | 17653 | 2.53006695194611 | 0.0214561680076414 | 0.599620100147433 | 1.66844783883504 | 0.0414201183431953 | CARHSP1//PSMB8//YWHAB//NUP214//SRSF3//TOB1//BTG2// |
| GO:0006351 | transcription, DNA-templated | Biological process | 49 | 3907 | 169 | 17653 | 1.31003978597056 | 0.0217722657113776 | 0.59973950075762 | 1.66209637413997 | 0.289940828402367 | OSR1//EP300//FOXO1//HEYL//ARHGAP35//KCNIP3//APBB1//SP5//FOXP3//BTG2//SRF//POLR2E//PAX8//CREB3L3//POLR3H//SRSF3//BAHD1//KMT2D//HAT1//ZNF618//ATP1B4//MYCBP//PFDN1//ASF1B//PRKCQ//ZNF395//HDAC11//DYNLL1//CARHSP1//HOXC9//ARID4B//PURA//OGT//MICAL2//NDP//ATXN7L3//TP53INP2//GPATCH3//SFRP1//YWHAB//BTAF1//TOB1//CTDSPL2//LMO2//MCIDAS//CAMK2A//PSMB8//PRKAA1//NPNT// |
| GO:0006688 | glycosphingolipid biosynthetic process | Biological process | 2 | 24 | 169 | 17653 | 8.70463510848126 | 0.0219038380433435 | 0.59973950075762 | 1.65947978036523 | 0.0118343195266272 | ST6GALNAC6//PRKAA1// |
| GO:0034695 | response to prostaglandin E | Biological process | 2 | 24 | 169 | 17653 | 8.70463510848126 | 0.0219038380433435 | 0.59973950075762 | 1.65947978036523 | 0.0118343195266272 | PRKAA1//SFRP1// |
| GO:0097186 | amelogenesis | Biological process | 2 | 24 | 169 | 17653 | 8.70463510848126 | 0.0219038380433435 | 0.59973950075762 | 1.65947978036523 | 0.0118343195266272 | FOXO1//ENAM// |
| GO:1903792 | negative regulation of anion transport | Biological process | 2 | 24 | 169 | 17653 | 8.70463510848126 | 0.0219038380433435 | 0.59973950075762 | 1.65947978036523 | 0.0118343195266272 | CYP4F2//OSR1// |
| GO:0044087 | regulation of cellular component biogenesis | Biological process | 15 | 881 | 169 | 17653 | 1.77847255337869 | 0.0221394217928821 | 0.603745603166378 | 1.65483372561808 | 0.0887573964497041 | TWF1//BRK1//EP300//TRABD2B//ARFIP2//ARHGAP35//PRKAA1//SRF//SFRP1//SPTBN5//BDNF//ABCA1//DYNLL1//NUDT16//SHQ1// |
| GO:0048167 | regulation of synaptic plasticity | Biological process | 5 | 168 | 169 | 17653 | 3.10879825302902 | 0.022878712053384 | 0.621400520550345 | 1.64056842759982 | 0.029585798816568 | CNTN2//CAMK2A//GRIN2B//SRF//RIMS4// |
| GO:0060218 | hematopoietic stem cell differentiation | Biological process | 4 | 113 | 169 | 17653 | 3.697544116877 | 0.0233272400993229 | 0.622521508763283 | 1.63213664056917 | 0.0236686390532544 | LMO2//PSMB8//SFRP1//SRF// |
| GO:0097659 | nucleic acid-templated transcription | Biological process | 49 | 3924 | 169 | 17653 | 1.30436428231065 | 0.0234537141697894 | 0.622521508763283 | 1.62978837189493 | 0.289940828402367 | OSR1//EP300//FOXO1//HEYL//ARHGAP35//KCNIP3//APBB1//SP5//FOXP3//BTG2//SRF//POLR2E//PAX8//CREB3L3//POLR3H//SRSF3//BAHD1//KMT2D//HAT1//ZNF618//ATP1B4//MYCBP//HOXC9//MCIDAS//ARID4B//PRKAA1//ASF1B//ZNF395//ATXN7L3//PURA//TP53INP2//GPATCH3//HDAC11//DYNLL1//PFDN1//PRKCQ//CARHSP1//OGT//MICAL2//NDP//SFRP1//YWHAB//BTAF1//TOB1//CTDSPL2//LMO2//CAMK2A//PSMB8//NPNT// |
| GO:0007264 | small GTPase mediated signal transduction | Biological process | 11 | 578 | 169 | 17653 | 1.98790974795766 | 0.0234826108790201 | 0.622521508763283 | 1.62925361835653 | 0.0650887573964497 | RHOF//BRK1//RABL3//RAB3B//ABCA1//OGT//ARHGEF6//ARHGAP35//RALBP1//CGNL1//ARFIP2// |
| GO:0009299 | mRNA transcription | Biological process | 2 | 25 | 169 | 17653 | 8.35644970414201 | 0.0236608688862389 | 0.622521508763283 | 1.62596931103648 | 0.0118343195266272 | LMO2//SRF// |
| GO:0030878 | thyroid gland development | Biological process | 2 | 25 | 169 | 17653 | 8.35644970414201 | 0.0236608688862389 | 0.622521508763283 | 1.62596931103648 | 0.0118343195266272 | SRF//PAX8// |
| GO:0034035 | purine ribonucleoside bisphosphate metabolic process | Biological process | 2 | 25 | 169 | 17653 | 8.35644970414201 | 0.0236608688862389 | 0.622521508763283 | 1.62596931103648 | 0.0118343195266272 | SULT4A1//SLC26A1// |
| GO:0043372 | positive regulation of CD4-positive, alpha-beta T cell differentiation | Biological process | 2 | 25 | 169 | 17653 | 8.35644970414201 | 0.0236608688862389 | 0.622521508763283 | 1.62596931103648 | 0.0118343195266272 | FOXP3//IL4R// |
| GO:0050427 | 3'-phosphoadenosine 5'-phosphosulfate metabolic process | Biological process | 2 | 25 | 169 | 17653 | 8.35644970414201 | 0.0236608688862389 | 0.622521508763283 | 1.62596931103648 | 0.0118343195266272 | SLC26A1//SULT4A1// |
| GO:0019538 | protein metabolic process | Biological process | 71 | 6078 | 169 | 17653 | 1.22019564205759 | 0.0237484177526138 | 0.622521508763283 | 1.62436532011067 | 0.420118343195266 | CDKN2D//GRIN2B//NCAM1//SPTBN5//PSMB8//YWHAB//CAMK2A//PRKAA1//UBE4B//MGRN1//COPS7B//ABHD17B//TULP4//CHRM1//STK35//PTPRT//PTPN18//CTDSPL2//LHPP//EP300//FOXO1//HAT1//BTG2//OGT//ABCA1//SPRN//PYURF//NUDT16//TRABD2B//ADAM11//SFRP1//CASP7//PRKCQ//TP53INP2//ARHGEF6//ST6GALNAC6//ATXN7L3//CRIM1//CNTN2//B4GALT5//ARID4B//HDAC11//USP54//NUP214//TOB1//PURA//MEX3A//OSR1//RAB3B//ZDHHC22//GPC1//CSPG5//FAF2//FOXP3//MIF//PPP1R26//GPD1L//SMPD1//UBXN2B//ARMC8//TWF1//ENAM//NUCB1//APBB1//KMT2D//POLR2E//SH3BP5//NPNT//ISCU//FAM161A//EFNA3// |
| GO:0006940 | regulation of smooth muscle contraction | Biological process | 3 | 65 | 169 | 17653 | 4.82102867546655 | 0.0244233761472128 | 0.635289588013847 | 1.61219430186708 | 0.0177514792899408 | CHRM1//NPNT//SRF// |
| GO:0072171 | mesonephric tubule morphogenesis | Biological process | 3 | 65 | 169 | 17653 | 4.82102867546655 | 0.0244233761472128 | 0.635289588013847 | 1.61219430186708 | 0.0177514792899408 | NPNT//PAX8//OSR1// |
| GO:0032774 | RNA biosynthetic process | Biological process | 49 | 3940 | 169 | 17653 | 1.29906737151954 | 0.025131131891268 | 0.639860192626217 | 1.59978795060116 | 0.289940828402367 | OSR1//EP300//FOXO1//HEYL//ARHGAP35//KCNIP3//APBB1//SP5//FOXP3//BTG2//SRF//POLR2E//PAX8//CREB3L3//POLR3H//SRSF3//BAHD1//KMT2D//HAT1//ZNF618//ATP1B4//MYCBP//HOXC9//MCIDAS//ARID4B//PRKAA1//ASF1B//ZNF395//ATXN7L3//PURA//TP53INP2//GPATCH3//HDAC11//DYNLL1//PFDN1//PRKCQ//CARHSP1//OGT//MICAL2//NDP//SFRP1//YWHAB//BTAF1//TOB1//CTDSPL2//LMO2//CAMK2A//PSMB8//NPNT// |
| GO:0065009 | regulation of molecular function | Biological process | 43 | 3373 | 169 | 17653 | 1.33163110464759 | 0.0252525545001317 | 0.639860192626217 | 1.59769468292367 | 0.254437869822485 | CDKN2D//PRKAA1//CASP7//CSPG5//MIF//NDP//BDNF//NPNT//PPP1R26//CRIM1//SH3BP4//FOXP3//EP300//RALBP1//ARHGAP35//GNAO1//SFRP1//ARHGEF6//BTAF1//YWHAB//FAF2//SMPD1//UBXN2B//DDAH1//SRF//PRKCQ//CAMK2A//PSMB8//SH3BP5//LYPD6//EFNA3//OSR1//CNIH2//DYNLL1//GPD1L//SCN3B//HEYL//GRIN2B//DENND6A//KCNIP3//NCAM1//SPTBN5//ST5// |
| GO:1903507 | negative regulation of nucleic acid-templated transcription | Biological process | 19 | 1224 | 169 | 17653 | 1.62145163785435 | 0.0253086212724763 | 0.639860192626217 | 1.59673151308216 | 0.112426035502959 | OSR1//EP300//FOXO1//HEYL//ARHGAP35//KCNIP3//APBB1//SP5//FOXP3//BTG2//BAHD1//KMT2D//HAT1//PURA//SFRP1//YWHAB//BTAF1//SRF//TOB1// |
| GO:0006513 | protein monoubiquitination | Biological process | 3 | 66 | 169 | 17653 | 4.74798278644433 | 0.0254109969877858 | 0.639860192626217 | 1.59497829528809 | 0.0177514792899408 | ATXN7L3//UBE4B//MGRN1// |
| GO:0032620 | interleukin-17 production | Biological process | 2 | 26 | 169 | 17653 | 8.03504779244424 | 0.0254737704460087 | 0.639860192626217 | 1.5939067691037 | 0.0118343195266272 | FOXP3//PRKCQ// |
| GO:0060343 | trabecula formation | Biological process | 2 | 26 | 169 | 17653 | 8.03504779244424 | 0.0254737704460087 | 0.639860192626217 | 1.5939067691037 | 0.0118343195266272 | SFRP1//SRF// |
| GO:0072202 | cell differentiation involved in metanephros development | Biological process | 2 | 26 | 169 | 17653 | 8.03504779244424 | 0.0254737704460087 | 0.639860192626217 | 1.5939067691037 | 0.0118343195266272 | PAX8//OSR1// |
| GO:1903827 | regulation of cellular protein localization | Biological process | 10 | 511 | 169 | 17653 | 2.04414131706018 | 0.0254794388387753 | 0.639860192626217 | 1.5938101411682 | 0.0591715976331361 | CTDSPL2//CHRM1//YWHAB//DYNLL1//ABHD17B//NUCB1//TP53INP2//PRKAA1//BDNF//GPD1L// |
| GO:0015031 | protein transport | Biological process | 27 | 1914 | 169 | 17653 | 1.47351189924134 | 0.0255205447526515 | 0.639860192626217 | 1.59311005955332 | 0.159763313609467 | SPRN//NUP214//SRSF3//YWHAB//ZDHHC22//GOSR2//TOMM40//ARFIP2//RABL3//KCNIP3//RAB3B//MLPH//CPLX1//FAF2//DYNLL1//SFRP1//CTDSPL2//ABCA1//FOXP3//APBB1//MIF//IL4R//CHRM1//NUCB1//PRKAA1//VPS37D//VPS53// |
| GO:0030029 | actin filament-based process | Biological process | 13 | 739 | 169 | 17653 | 1.83751431248048 | 0.0255452095237437 | 0.639860192626217 | 1.59269053085087 | 0.0769230769230769 | RHOF//TWF1//MICAL2//ARFIP2//SPTBN5//BRK1//CGNL1//ARHGAP35//MLPH//SRF//SFRP1//SCN3B//GPD1L// |
| GO:1902679 | negative regulation of RNA biosynthetic process | Biological process | 19 | 1226 | 169 | 17653 | 1.6188065291466 | 0.0256838384653939 | 0.640958669894682 | 1.59034007017622 | 0.112426035502959 | OSR1//EP300//FOXO1//HEYL//ARHGAP35//KCNIP3//APBB1//SP5//FOXP3//BTG2//BAHD1//KMT2D//HAT1//PURA//SFRP1//YWHAB//BTAF1//SRF//TOB1// |
| GO:0090101 | negative regulation of transmembrane receptor protein serine/threonine kinase signaling pathway | Biological process | 4 | 117 | 169 | 17653 | 3.57113235219744 | 0.0260909496907203 | 0.647382483374215 | 1.58351011262332 | 0.0236686390532544 | FAM89B//TOB1//CTDSPL2//SFRP1// |
| GO:0003281 | ventricular septum development | Biological process | 3 | 67 | 169 | 17653 | 4.67711737172128 | 0.0264198677230938 | 0.647382483374215 | 1.57806936110735 | 0.0177514792899408 | HEYL//FGFRL1//PAX8// |
| GO:0006110 | regulation of glycolytic process | Biological process | 3 | 67 | 169 | 17653 | 4.67711737172128 | 0.0264198677230938 | 0.647382483374215 | 1.57806936110735 | 0.0177514792899408 | PRKAA1//NUP214//OGT// |
| GO:0030521 | androgen receptor signaling pathway | Biological process | 3 | 67 | 169 | 17653 | 4.67711737172128 | 0.0264198677230938 | 0.647382483374215 | 1.57806936110735 | 0.0177514792899408 | EP300//HEYL//SFRP1// |
| GO:1902108 | regulation of mitochondrial membrane permeability involved in apoptotic process | Biological process | 3 | 67 | 169 | 17653 | 4.67711737172128 | 0.0264198677230938 | 0.647382483374215 | 1.57806936110735 | 0.0177514792899408 | YWHAB//DYNLL1//CAMK2A// |
| GO:0009266 | response to temperature stimulus | Biological process | 6 | 237 | 169 | 17653 | 2.6444461089057 | 0.0267164032779889 | 0.652285326242018 | 1.57322200958208 | 0.0355029585798817 | PRKAA1//ANO3//FOXO1//EP300//NUP214//CAMK2A// |
| GO:0032673 | regulation of interleukin-4 production | Biological process | 2 | 27 | 169 | 17653 | 7.73745342976112 | 0.0273413254288715 | 0.660390656698064 | 1.56318043590966 | 0.0118343195266272 | FOXP3//PRKCQ// |
| GO:0048643 | positive regulation of skeletal muscle tissue development | Biological process | 2 | 27 | 169 | 17653 | 7.73745342976112 | 0.0273413254288715 | 0.660390656698064 | 1.56318043590966 | 0.0118343195266272 | GPC1//PRKAA1// |
| GO:0072207 | metanephric epithelium development | Biological process | 2 | 27 | 169 | 17653 | 7.73745342976112 | 0.0273413254288715 | 0.660390656698064 | 1.56318043590966 | 0.0118343195266272 | PAX8//OSR1// |
| GO:0009247 | glycolipid biosynthetic process | Biological process | 3 | 68 | 169 | 17653 | 4.60833623390184 | 0.0274499645571203 | 0.660655196796458 | 1.56145821196642 | 0.0177514792899408 | ST6GALNAC6//PYURF//PRKAA1// |
| GO:0021954 | central nervous system neuron development | Biological process | 3 | 69 | 169 | 17653 | 4.54154875225109 | 0.0285012578073333 | 0.66743066819239 | 1.54513597343955 | 0.0177514792899408 | CNTN2//ARHGAP35//BTG2// |
| GO:0035914 | skeletal muscle cell differentiation | Biological process | 3 | 69 | 169 | 17653 | 4.54154875225109 | 0.0285012578073333 | 0.66743066819239 | 1.54513597343955 | 0.0177514792899408 | GPC1//HEYL//BTG2// |
| GO:0071310 | cellular response to organic substance | Biological process | 35 | 2656 | 169 | 17653 | 1.37648597347972 | 0.028573346733576 | 0.66743066819239 | 1.5440388886757 | 0.207100591715976 | EP300//CHRM1//FOXO1//FGFRL1//POLR2E//IL4R//SH3BP4//TOB1//NPNT//FAM89B//CTDSPL2//SFRP1//CREB3L3//MIF//KMT2D//PSMB8//DYNLL1//GOSR2//CAMK2A//BDNF//GPC1//OGT//PRKCQ//NCAM1//HEYL//ABCA1//OSR1//SRF//PTPN18//PRKAA1//PAX8//SP5//CASP7//LMO2//SRSF3// |
| GO:0009966 | regulation of signal transduction | Biological process | 41 | 3215 | 169 | 17653 | 1.33209345983601 | 0.028896890975606 | 0.66743066819239 | 1.53914888059196 | 0.242603550295858 | PRKAA1//TOB1//CSPG5//MIF//NDP//BDNF//MGRN1//YWHAB//SMPD1//FOXP3//SFRP1//TRABD2B//NPNT//FAM89B//CTDSPL2//SH3BP4//ABCA1//FOXO1//KMT2D//OGT//ARHGEF6//ARHGAP35//GPATCH3//GPC1//VAPA//EP300//EVC//PRKCQ//P2RX2//RALBP1//RHOF//CGNL1//CNTN2//HEYL//GPD1L//PSMB8//LYPD6//DYNLL1//PTPN18//CDKN2D//CNIH2// |
| GO:0003231 | cardiac ventricle development | Biological process | 4 | 121 | 169 | 17653 | 3.45307839014133 | 0.0290434771702986 | 0.66743066819239 | 1.53695138984963 | 0.0236686390532544 | HEYL//UBE4B//PAX8//FGFRL1// |
| GO:0009135 | purine nucleoside diphosphate metabolic process | Biological process | 4 | 121 | 169 | 17653 | 3.45307839014133 | 0.0290434771702986 | 0.66743066819239 | 1.53695138984963 | 0.0236686390532544 | NUP214//OGT//PRKAA1//NUDT16// |
| GO:0009179 | purine ribonucleoside diphosphate metabolic process | Biological process | 4 | 121 | 169 | 17653 | 3.45307839014133 | 0.0290434771702986 | 0.66743066819239 | 1.53695138984963 | 0.0236686390532544 | NUP214//OGT//PRKAA1//NUDT16// |
| GO:0046636 | negative regulation of alpha-beta T cell activation | Biological process | 2 | 28 | 169 | 17653 | 7.46111580726965 | 0.0292623344846297 | 0.66743066819239 | 1.53369102977085 | 0.0118343195266272 | IL4R//FOXP3// |
| GO:0048841 | regulation of axon extension involved in axon guidance | Biological process | 2 | 28 | 169 | 17653 | 7.46111580726965 | 0.0292623344846297 | 0.66743066819239 | 1.53369102977085 | 0.0118343195266272 | SEMA4B//SEMA4G// |
| GO:0051125 | regulation of actin nucleation | Biological process | 2 | 28 | 169 | 17653 | 7.46111580726965 | 0.0292623344846297 | 0.66743066819239 | 1.53369102977085 | 0.0118343195266272 | ARFIP2//BRK1// |
| GO:1902803 | regulation of synaptic vesicle transport | Biological process | 2 | 28 | 169 | 17653 | 7.46111580726965 | 0.0292623344846297 | 0.66743066819239 | 1.53369102977085 | 0.0118343195266272 | CNIH2//RIMS4// |
| GO:0048522 | positive regulation of cellular process | Biological process | 61 | 5142 | 169 | 17653 | 1.23916625815019 | 0.0293208133785611 | 0.66743066819239 | 1.53282398625052 | 0.36094674556213 | PRKAA1//SRF//EP300//BTG2//HEYL//CHRM1//NUDT16//BRK1//PURA//SFRP1//KMT2D//CASP7//TOB1//FOXO1//SH3BP4//TP53INP2//P2RX2//CAMK2A//NPNT//MICAL2//MIF//CNTN2//ARHGAP35//APBB1//TWF1//NDP//FOXP3//ATXN7L3//GPATCH3//PAX8//SEMA4B//FAM89B//SEMA4G//FERMT3//BDNF//PRKCQ//TRABD2B//ARFIP2//SMPD1//DYNLL1//IL4R//SHQ1//ARHGEF6//VAPA//DDAH1//OGT//EVC//OSR1//ATP1B4//MCIDAS//LMO2//ARID4B//CREB3L3//RIMS4//RAB3B//GRIN2B//PSMB8//YWHAB//FAM161A//EFNA3//GPC1// |
| GO:0070482 | response to oxygen levels | Biological process | 8 | 378 | 169 | 17653 | 2.21070097993175 | 0.0293427891772641 | 0.66743066819239 | 1.53249860669172 | 0.0473372781065089 | EP300//P2RX2//PRKAA1//SRF//PSMB8//FOXO1//SFRP1//DDAH1// |
| GO:0006886 | intracellular protein transport | Biological process | 17 | 1078 | 169 | 17653 | 1.64725933407252 | 0.029490173535631 | 0.66743066819239 | 1.53032267182442 | 0.100591715976331 | SPRN//NUP214//SRSF3//YWHAB//ZDHHC22//GOSR2//TOMM40//FAF2//CTDSPL2//CHRM1//NUCB1//PRKAA1//ARFIP2//RABL3//KCNIP3//RAB3B//MLPH// |
| GO:0006479 | protein methylation | Biological process | 5 | 180 | 169 | 17653 | 2.90154503616042 | 0.0296435867107247 | 0.66743066819239 | 1.52806925027313 | 0.029585798816568 | RAB3B//ARID4B//KMT2D//OGT//BTG2// |
| GO:0008213 | protein alkylation | Biological process | 5 | 180 | 169 | 17653 | 2.90154503616042 | 0.0296435867107247 | 0.66743066819239 | 1.52806925027313 | 0.029585798816568 | BTG2//RAB3B//ARID4B//KMT2D//OGT// |
| GO:0050896 | response to stimulus | Biological process | 100 | 9123 | 169 | 17653 | 1.14497008990217 | 0.0296749426321457 | 0.66743066819239 | 1.52761011195439 | 0.591715976331361 | GRIN2B//NCAM1//SPTBN5//PSMB8//YWHAB//CAMK2A//PRKAA1//COPS7B//CDKN2D//EP300//P2RX2//SRF//FOXP3//IL4R//CREB3L3//FAF2//CLEC1A//AKAP10//CYP4F2//TRABD2B//NDP//GNAO1//RALBP1//PTPRT//CHRM1//ARHGAP35//KCNIP3//APBB1//FGFRL1//SMPD1//OGT//VAPA//SH3BP5//BDNF//CARHSP1//PRKCQ//BTG2//POLR2E//MIF//FOXO1//ABCA1//GNAZ//GPR153//SFRP1//ARHGEF6//GPR3//HEYL//EVC//ADAM11//FERMT3//OSR1//DDAH1//ARFIP2//RHOF//EFNA3//GPC1//CNTN2//UNC5A//HAT1//UBE4B//TOB1//CSPG5//TWF1//ANO3//MGRN1//BRK1//SH3BP4//NPNT//FAM89B//CTDSPL2//RABL3//RAB3B//KMT2D//GPX8//DYNLL1//GOSR2//PTPN18//PAX8//GPATCH3//ARMC8//POLR3H//CRIM1//SEMA4B//SEMA4G//BEST1//CGNL1//SP5//ZNF385B//NUCB1//CASP7//GPD1L//LMO2//BSN//LYPD6//NUP214//ZSWIM6//SRSF3//CNIH2//NUDT16//FAM161A// |
| GO:0014706 | striated muscle tissue development | Biological process | 8 | 379 | 169 | 17653 | 2.20486799581583 | 0.0297402166170669 | 0.66743066819239 | 1.52665587255737 | 0.0473372781065089 | UBE4B//EP300//HEYL//BTG2//PRKAA1//P2RX2//SRF//GPC1// |
| GO:0001838 | embryonic epithelial tube formation | Biological process | 4 | 122 | 169 | 17653 | 3.42477446891066 | 0.0298113479763376 | 0.66743066819239 | 1.52561838607909 | 0.0236686390532544 | ARHGAP35//OSR1//PAX8//SFRP1// |
| GO:0071702 | organic substance transport | Biological process | 35 | 2667 | 169 | 17653 | 1.37080867850099 | 0.030165834505742 | 0.66743066819239 | 1.52048465588625 | 0.207100591715976 | SPRN//NUP214//SRSF3//YWHAB//ZDHHC22//GOSR2//TOMM40//ABCA1//PCTP//ANO3//ARFIP2//RABL3//KCNIP3//RAB3B//MLPH//CPLX1//VPS37D//VPS53//SLC26A1//BEST1//FAF2//CYP4F2//DYNLL1//SFRP1//CTDSPL2//FOXP3//APBB1//MIF//IL4R//CHRM1//RALBP1//NUCB1//PRKAA1//OSR1//PAX8// |
| GO:0045926 | negative regulation of growth | Biological process | 6 | 244 | 169 | 17653 | 2.568580851683 | 0.0302092025898925 | 0.66743066819239 | 1.51986073832752 | 0.0355029585798817 | CDKN2D//SH3BP4//APBB1//SFRP1//SEMA4B//SEMA4G// |
| GO:0008064 | regulation of actin polymerization or depolymerization | Biological process | 5 | 181 | 169 | 17653 | 2.88551440060152 | 0.0302584239004609 | 0.66743066819239 | 1.5191536972368 | 0.029585798816568 | TWF1//ARFIP2//SPTBN5//BRK1//ARHGAP35// |
| GO:0009185 | ribonucleoside diphosphate metabolic process | Biological process | 4 | 123 | 169 | 17653 | 3.39693077404147 | 0.030591167551149 | 0.66743066819239 | 1.51440394729303 | 0.0236686390532544 | NUP214//OGT//PRKAA1//NUDT16// |
| GO:0032479 | regulation of type I interferon production | Biological process | 4 | 123 | 169 | 17653 | 3.39693077404147 | 0.030591167551149 | 0.66743066819239 | 1.51440394729303 | 0.0236686390532544 | GPATCH3//POLR3H//EP300//POLR2E// |
| GO:0060359 | response to ammonium ion | Biological process | 4 | 123 | 169 | 17653 | 3.39693077404147 | 0.030591167551149 | 0.66743066819239 | 1.51440394729303 | 0.0236686390532544 | CHRM1//SMPD1//GNAO1//CASP7// |
| GO:0030811 | regulation of nucleotide catabolic process | Biological process | 3 | 71 | 169 | 17653 | 4.41361780148346 | 0.0306672863158106 | 0.66743066819239 | 1.51332465213003 | 0.0177514792899408 | NUP214//OGT//PRKAA1// |
| GO:0090114 | COPII-coated vesicle budding | Biological process | 3 | 71 | 169 | 17653 | 4.41361780148346 | 0.0306672863158106 | 0.66743066819239 | 1.51332465213003 | 0.0177514792899408 | CNIH2//GOSR2//VAPA// |
| GO:1900076 | regulation of cellular response to insulin stimulus | Biological process | 3 | 71 | 169 | 17653 | 4.41361780148346 | 0.0306672863158106 | 0.66743066819239 | 1.51332465213003 | 0.0177514792899408 | OGT//PRKCQ//PRKAA1// |
| GO:0009887 | animal organ morphogenesis | Biological process | 16 | 1001 | 169 | 17653 | 1.66962032050789 | 0.0307703865783287 | 0.66743066819239 | 1.51186704753609 | 0.0946745562130177 | NPNT//PAX8//SRF//MICAL2//HEYL//UBE4B//OSR1//TMIE//HOXC9//PSMB8//SFRP1//SP5//FGFRL1//FOXO1//ENAM//EP300// |
| GO:0030832 | regulation of actin filament length | Biological process | 5 | 182 | 169 | 17653 | 2.86965992587294 | 0.0308812541581618 | 0.66743066819239 | 1.51030507028874 | 0.029585798816568 | TWF1//ARHGAP35//BRK1//ARFIP2//SPTBN5// |
| GO:0001782 | B cell homeostasis | Biological process | 2 | 29 | 169 | 17653 | 7.20383595184656 | 0.0312356159780302 | 0.66743066819239 | 1.50534992518389 | 0.0118343195266272 | MIF//FOXP3// |
| GO:0032892 | positive regulation of organic acid transport | Biological process | 2 | 29 | 169 | 17653 | 7.20383595184656 | 0.0312356159780302 | 0.66743066819239 | 1.50534992518389 | 0.0118343195266272 | CYP4F2//MIF// |
| GO:0070207 | protein homotrimerization | Biological process | 2 | 29 | 169 | 17653 | 7.20383595184656 | 0.0312356159780302 | 0.66743066819239 | 1.50534992518389 | 0.0118343195266272 | MIF//BRK1// |
| GO:1902230 | negative regulation of intrinsic apoptotic signaling pathway in response to DNA damage | Biological process | 2 | 29 | 169 | 17653 | 7.20383595184656 | 0.0312356159780302 | 0.66743066819239 | 1.50534992518389 | 0.0118343195266272 | MIF//CDKN2D// |
| GO:2000272 | negative regulation of signaling receptor activity | Biological process | 2 | 29 | 169 | 17653 | 7.20383595184656 | 0.0312356159780302 | 0.66743066819239 | 1.50534992518389 | 0.0118343195266272 | HEYL//LYPD6// |
| GO:0003206 | cardiac chamber morphogenesis | Biological process | 4 | 124 | 169 | 17653 | 3.369536171025 | 0.0313829591135857 | 0.66743066819239 | 1.5033061089928 | 0.0236686390532544 | HEYL//UBE4B//SRF//FGFRL1// |
| GO:0032606 | type I interferon production | Biological process | 4 | 124 | 169 | 17653 | 3.369536171025 | 0.0313829591135857 | 0.66743066819239 | 1.5033061089928 | 0.0236686390532544 | GPATCH3//POLR3H//EP300//POLR2E// |
| GO:0050794 | regulation of cellular process | Biological process | 118 | 11074 | 169 | 17653 | 1.11303623926399 | 0.0318368899015679 | 0.671253528398831 | 1.49706936444439 | 0.698224852071006 | CDKN2D//OSR1//EP300//FOXO1//HEYL//ARHGAP35//KCNIP3//APBB1//SP5//FOXP3//BTG2//GRIN2B//NCAM1//SPTBN5//PSMB8//YWHAB//CAMK2A//PRKAA1//CRIM1//PRKCQ//MIF//SRF//NUP214//TWF1//TRABD2B//NDP//GNAO1//RALBP1//PTPRT//AKAP10//CHRM1//IL4R//FGFRL1//SMPD1//OGT//VAPA//SH3BP5//BDNF//CARHSP1//MCIDAS//BAHD1//KMT2D//HAT1//ZNF618//ATP1B4//MYCBP//PFDN1//ASF1B//ZNF395//HDAC11//DYNLL1//HOXC9//ARID4B//PURA//CLEC1A//ABCA1//GNAZ//GPR153//SFRP1//ARHGEF6//GPR3//EVC//ADAM11//FERMT3//DDAH1//ARFIP2//RHOF//TTC28//BRK1//NUDT16//TOB1//SH3BP4//POLR2E//UBE4B//CASP7//CSPG5//TP53INP2//P2RX2//PAX8//NPNT//MICAL2//PPP1R26//CNTN2//MGRN1//ATXN7L3//GPATCH3//BTAF1//MEX3A//CPLX1//RAB3B//ENAM//KIF2A//SEMA4B//FAM89B//SEMA4G//CTDSPL2//CREB3L3//RABL3//GPD1L//GOSR2//UNC5A//PTPN18//GPC1//SHQ1//UBXN2B//EFNA3//LMO2//SRSF3//RIMS4//CGNL1//SCN3B//ZNF385B//BSN//LYPD6//FAM161A//ZSWIM6//ABHD17B//CNIH2// |
| GO:0060562 | epithelial tube morphogenesis | Biological process | 7 | 316 | 169 | 17653 | 2.31389034529249 | 0.032692070874701 | 0.671253528398831 | 1.48555756822448 | 0.0414201183431953 | SRF//NPNT//PAX8//ARHGAP35//MICAL2//OSR1//SFRP1// |
| GO:0045892 | negative regulation of transcription, DNA-templated | Biological process | 18 | 1176 | 169 | 17653 | 1.59881053012921 | 0.0328428800665711 | 0.671253528398831 | 1.48355876561547 | 0.106508875739645 | OSR1//EP300//FOXO1//HEYL//ARHGAP35//KCNIP3//APBB1//SP5//FOXP3//BTG2//BAHD1//KMT2D//HAT1//SRF//PURA//SFRP1//YWHAB//BTAF1// |
| GO:0006283 | transcription-coupled nucleotide-excision repair | Biological process | 3 | 73 | 169 | 17653 | 4.29269676582638 | 0.032917603847572 | 0.671253528398831 | 1.48257178566061 | 0.0177514792899408 | EP300//POLR2E//COPS7B// |
| GO:0006687 | glycosphingolipid metabolic process | Biological process | 3 | 73 | 169 | 17653 | 4.29269676582638 | 0.032917603847572 | 0.671253528398831 | 1.48257178566061 | 0.0177514792899408 | ST6GALNAC6//PRKAA1//SMPD1// |
| GO:0032481 | positive regulation of type I interferon production | Biological process | 3 | 73 | 169 | 17653 | 4.29269676582638 | 0.032917603847572 | 0.671253528398831 | 1.48257178566061 | 0.0177514792899408 | POLR3H//EP300//POLR2E// |
| GO:0035019 | somatic stem cell population maintenance | Biological process | 3 | 73 | 169 | 17653 | 4.29269676582638 | 0.032917603847572 | 0.671253528398831 | 1.48257178566061 | 0.0177514792899408 | PAX8//POLR2E//SFRP1// |
| GO:0035710 | CD4-positive, alpha-beta T cell activation | Biological process | 3 | 73 | 169 | 17653 | 4.29269676582638 | 0.032917603847572 | 0.671253528398831 | 1.48257178566061 | 0.0177514792899408 | FOXP3//IL4R//PRKCQ// |
| GO:0072078 | nephron tubule morphogenesis | Biological process | 3 | 73 | 169 | 17653 | 4.29269676582638 | 0.032917603847572 | 0.671253528398831 | 1.48257178566061 | 0.0177514792899408 | NPNT//PAX8//OSR1// |
| GO:0034654 | nucleobase-containing compound biosynthetic process | Biological process | 54 | 4488 | 169 | 17653 | 1.25681897288232 | 0.0329571084198175 | 0.671253528398831 | 1.48205089929306 | 0.319526627218935 | OSR1//EP300//FOXO1//HEYL//ARHGAP35//KCNIP3//APBB1//SP5//FOXP3//BTG2//CDKN2D//SRF//POLR2E//PAX8//CREB3L3//POLR3H//SRSF3//NUP214//OGT//BAHD1//KMT2D//HAT1//ZNF618//ATP1B4//MYCBP//HOXC9//MCIDAS//ARID4B//PRKAA1//ASF1B//ZNF395//ATXN7L3//PURA//TP53INP2//GPATCH3//HDAC11//DYNLL1//PFDN1//PRKCQ//CARHSP1//LHPP//MICAL2//NDP//SFRP1//YWHAB//BTAF1//TOB1//CTDSPL2//NUDT16//LMO2//SLC26A1//CAMK2A//PSMB8//NPNT// |
| GO:0006664 | glycolipid metabolic process | Biological process | 4 | 126 | 169 | 17653 | 3.31605146989762 | 0.0330025415341583 | 0.671253528398831 | 1.48145261370519 | 0.0236686390532544 | ST6GALNAC6//PYURF//SMPD1//PRKAA1// |
| GO:1903509 | liposaccharide metabolic process | Biological process | 4 | 126 | 169 | 17653 | 3.31605146989762 | 0.0330025415341583 | 0.671253528398831 | 1.48145261370519 | 0.0236686390532544 | ST6GALNAC6//PYURF//SMPD1//PRKAA1// |
| GO:0045622 | regulation of T-helper cell differentiation | Biological process | 2 | 30 | 169 | 17653 | 6.96370808678501 | 0.0332600057628174 | 0.671253528398831 | 1.47807767986817 | 0.0118343195266272 | IL4R//FOXP3// |
| GO:1900739 | regulation of protein insertion into mitochondrial membrane involved in apoptotic signaling pathway | Biological process | 2 | 30 | 169 | 17653 | 6.96370808678501 | 0.0332600057628174 | 0.671253528398831 | 1.47807767986817 | 0.0118343195266272 | YWHAB//DYNLL1// |
| GO:1900740 | positive regulation of protein insertion into mitochondrial membrane involved in apoptotic signaling pathway | Biological process | 2 | 30 | 169 | 17653 | 6.96370808678501 | 0.0332600057628174 | 0.671253528398831 | 1.47807767986817 | 0.0118343195266272 | YWHAB//DYNLL1// |
| GO:0007050 | cell cycle arrest | Biological process | 6 | 250 | 169 | 17653 | 2.5069349112426 | 0.0334315137805403 | 0.671253528398831 | 1.47584395808733 | 0.0355029585798817 | EP300//BTG2//MIF//CDKN2D//APBB1//PRKAA1// |
| GO:0051239 | regulation of multicellular organismal process | Biological process | 38 | 2969 | 169 | 17653 | 1.33691936997893 | 0.0338131592572771 | 0.671253528398831 | 1.47091425000018 | 0.224852071005917 | GPD1L//FOXP3//CHRM1//GNAO1//NPNT//SRF//SCN3B//TWF1//SFRP1//ARHGAP35//APBB1//EFNA3//OSR1//CNTN2//GPATCH3//POLR3H//EP300//POLR2E//PRKCQ//MIF//IL4R//KMT2D//BDNF//HEYL//TOB1//DDAH1//PRKAA1//SEMA4B//SEMA4G//PSMB8//KCNIP3//ENAM//PAX8//ABCA1//LMO2//ZSWIM6//FOXO1//GPC1// |
| GO:0035304 | regulation of protein dephosphorylation | Biological process | 4 | 127 | 169 | 17653 | 3.28994082840237 | 0.0338303693258753 | 0.671253528398831 | 1.4706932609967 | 0.0236686390532544 | PPP1R26//SMPD1//YWHAB//UBXN2B// |
| GO:0010557 | positive regulation of macromolecule biosynthetic process | Biological process | 24 | 1698 | 169 | 17653 | 1.47640454136785 | 0.0339495331595347 | 0.671253528398831 | 1.4691661933314 | 0.142011834319527 | SRF//EP300//HEYL//MICAL2//FOXO1//APBB1//NDP//FOXP3//ATXN7L3//TP53INP2//GPATCH3//SFRP1//PAX8//PRKCQ//OSR1//ATP1B4//MCIDAS//LMO2//ARID4B//KMT2D//CREB3L3//OGT//PRKAA1//NPNT// |
| GO:0014031 | mesenchymal cell development | Biological process | 3 | 74 | 169 | 17653 | 4.23468735007197 | 0.0340742385509751 | 0.671253528398831 | 1.46757384046188 | 0.0177514792899408 | SEMA4B//SEMA4G//HEYL// |
| GO:0046634 | regulation of alpha-beta T cell activation | Biological process | 3 | 74 | 169 | 17653 | 4.23468735007197 | 0.0340742385509751 | 0.671253528398831 | 1.46757384046188 | 0.0177514792899408 | FOXP3//IL4R//PRKCQ// |
| GO:0018205 | peptidyl-lysine modification | Biological process | 8 | 390 | 169 | 17653 | 2.14267941131846 | 0.0343594926133326 | 0.671253528398831 | 1.46395325802056 | 0.0473372781065089 | EP300//NUP214//ARID4B//FOXP3//APBB1//HAT1//OGT//KMT2D// |
| GO:0010628 | positive regulation of gene expression | Biological process | 26 | 1877 | 169 | 17653 | 1.4469079136101 | 0.0343618293748189 | 0.671253528398831 | 1.4639237230061 | 0.153846153846154 | SRF//EP300//HEYL//MICAL2//CNTN2//FOXO1//APBB1//NDP//FOXP3//ATXN7L3//TP53INP2//GPATCH3//SFRP1//PAX8//POLR2E//OSR1//ATP1B4//MCIDAS//LMO2//ARID4B//KMT2D//CREB3L3//OGT//TOB1//PRKAA1//NPNT// |
| GO:0051260 | protein homooligomerization | Biological process | 7 | 320 | 169 | 17653 | 2.28496671597633 | 0.0346375120554483 | 0.671253528398831 | 1.46045331005522 | 0.0414201183431953 | DPYS//MIF//BRK1//KCTD18//P2RX2//SPTBN5//FGFRL1// |
| GO:1901136 | carbohydrate derivative catabolic process | Biological process | 5 | 188 | 169 | 17653 | 2.77807503462168 | 0.0347874466345055 | 0.671253528398831 | 1.45857744688449 | 0.029585798816568 | GPC1//CSPG5//NUDT16//DPYS//GPD1L// |
| GO:0031667 | response to nutrient levels | Biological process | 9 | 465 | 169 | 17653 | 2.021721702615 | 0.0352276370644799 | 0.671253528398831 | 1.45311648665906 | 0.0532544378698225 | ABCA1//OGT//HAT1//FOXO1//SFRP1//PRKAA1//P2RX2//CDKN2D//SRF// |
| GO:0008589 | regulation of smoothened signaling pathway | Biological process | 3 | 75 | 169 | 17653 | 4.17822485207101 | 0.0352517764012877 | 0.671253528398831 | 1.45281899323036 | 0.0177514792899408 | MGRN1//EVC//SFRP1// |
| GO:0043470 | regulation of carbohydrate catabolic process | Biological process | 3 | 75 | 169 | 17653 | 4.17822485207101 | 0.0352517764012877 | 0.671253528398831 | 1.45281899323036 | 0.0177514792899408 | NUP214//OGT//PRKAA1// |
| GO:0072088 | nephron epithelium morphogenesis | Biological process | 3 | 75 | 169 | 17653 | 4.17822485207101 | 0.0352517764012877 | 0.671253528398831 | 1.45281899323036 | 0.0177514792899408 | NPNT//PAX8//OSR1// |
| GO:1905954 | positive regulation of lipid localization | Biological process | 3 | 75 | 169 | 17653 | 4.17822485207101 | 0.0352517764012877 | 0.671253528398831 | 1.45281899323036 | 0.0177514792899408 | ABCA1//CYP4F2//MIF// |
| GO:2001169 | regulation of ATP biosynthetic process | Biological process | 3 | 75 | 169 | 17653 | 4.17822485207101 | 0.0352517764012877 | 0.671253528398831 | 1.45281899323036 | 0.0177514792899408 | NUP214//OGT//PRKAA1// |
| GO:0006691 | leukotriene metabolic process | Biological process | 2 | 31 | 169 | 17653 | 6.73907234205001 | 0.035334356958448 | 0.671253528398831 | 1.45180280789925 | 0.0118343195266272 | CYP4F3//CYP4F2// |
| GO:0010765 | positive regulation of sodium ion transport | Biological process | 2 | 31 | 169 | 17653 | 6.73907234205001 | 0.035334356958448 | 0.671253528398831 | 1.45180280789925 | 0.0118343195266272 | GPD1L//SCN3B// |
| GO:0034694 | response to prostaglandin | Biological process | 2 | 31 | 169 | 17653 | 6.73907234205001 | 0.035334356958448 | 0.671253528398831 | 1.45180280789925 | 0.0118343195266272 | PRKAA1//SFRP1// |
| GO:0043368 | positive T cell selection | Biological process | 2 | 31 | 169 | 17653 | 6.73907234205001 | 0.035334356958448 | 0.671253528398831 | 1.45180280789925 | 0.0118343195266272 | FOXP3//SRF// |
| GO:0045880 | positive regulation of smoothened signaling pathway | Biological process | 2 | 31 | 169 | 17653 | 6.73907234205001 | 0.035334356958448 | 0.671253528398831 | 1.45180280789925 | 0.0118343195266272 | EVC//SFRP1// |
| GO:0050869 | negative regulation of B cell activation | Biological process | 2 | 31 | 169 | 17653 | 6.73907234205001 | 0.035334356958448 | 0.671253528398831 | 1.45180280789925 | 0.0118343195266272 | SFRP1//FOXP3// |
| GO:0070229 | negative regulation of lymphocyte apoptotic process | Biological process | 2 | 31 | 169 | 17653 | 6.73907234205001 | 0.035334356958448 | 0.671253528398831 | 1.45180280789925 | 0.0118343195266272 | MIF//PRKCQ// |
| GO:0010243 | response to organonitrogen compound | Biological process | 15 | 937 | 169 | 17653 | 1.67218177110633 | 0.0355079738469868 | 0.672662260860425 | 1.44967410866652 | 0.0887573964497041 | FOXO1//SH3BP4//UBE4B//FAF2//PRKAA1//OGT//P2RX2//CAMK2A//SMPD1//GNAO1//BTG2//PRKCQ//ABCA1//SFRP1//CASP7// |
| GO:0110053 | regulation of actin filament organization | Biological process | 6 | 254 | 169 | 17653 | 2.46745562130178 | 0.0356992221892003 | 0.674396200183133 | 1.44734124614602 | 0.0355029585798817 | TWF1//ARHGAP35//BRK1//ARFIP2//SFRP1//SPTBN5// |
| GO:0032870 | cellular response to hormone stimulus | Biological process | 12 | 698 | 169 | 17653 | 1.79579864702192 | 0.0363359666038705 | 0.675534243173841 | 1.43966328228114 | 0.0710059171597633 | CHRM1//FOXO1//KMT2D//CAMK2A//OGT//PRKCQ//EP300//HEYL//SFRP1//PAX8//PRKAA1//LMO2// |
| GO:0072175 | epithelial tube formation | Biological process | 4 | 130 | 169 | 17653 | 3.2140191169777 | 0.0363861813210035 | 0.675534243173841 | 1.43906352062257 | 0.0236686390532544 | ARHGAP35//OSR1//PAX8//SFRP1// |
| GO:0044260 | cellular macromolecule metabolic process | Biological process | 94 | 8555 | 169 | 17653 | 1.14772979571793 | 0.0363954168293997 | 0.675534243173841 | 1.43895330236802 | 0.556213017751479 | CDKN2D//OSR1//EP300//FOXO1//HEYL//ARHGAP35//KCNIP3//APBB1//SP5//FOXP3//BTG2//GRIN2B//NCAM1//SPTBN5//PSMB8//YWHAB//CAMK2A//PRKAA1//UBE4B//MGRN1//COPS7B//ABHD17B//SRF//POLR2E//PAX8//CREB3L3//POLR3H//SRSF3//TULP4//PURA//MCIDAS//BAHD1//KMT2D//HAT1//ZNF618//ATP1B4//MYCBP//HOXC9//ARID4B//ASF1B//ZNF395//ATXN7L3//TP53INP2//GPATCH3//HDAC11//DYNLL1//PFDN1//PRKCQ//CARHSP1//OGT//NUDT16//CHRM1//STK35//PTPRT//PTPN18//CTDSPL2//LHPP//ABCA1//SPRN//PYURF//ARHGEF6//CASP7//ST6GALNAC6//MICAL2//CRIM1//CNTN2//B4GALT5//NDP//SFRP1//BTAF1//USP54//NUP214//TOB1//MEX3A//RAB3B//ZDHHC22//GPC1//CSPG5//FAF2//MIF//PPP1R26//GPD1L//SMPD1//LMO2//UBXN2B//ARMC8//TWF1//ENAM//NUCB1//SH3BP5//NPNT//FAM161A//EFNA3//TRABD2B// |
| GO:1905114 | cell surface receptor signaling pathway involved in cell-cell signaling | Biological process | 11 | 620 | 169 | 17653 | 1.85324489406375 | 0.0364337656316172 | 0.675534243173841 | 1.43849593969644 | 0.0650887573964497 | TRABD2B//NDP//PRKAA1//GNAO1//CAMK2A//FOXP3//SFRP1//PSMB8//P2RX2//GRIN2B//FOXO1// |
| GO:0045652 | regulation of megakaryocyte differentiation | Biological process | 3 | 76 | 169 | 17653 | 4.1232482092806 | 0.0364501506757336 | 0.675534243173841 | 1.4383006720784 | 0.0177514792899408 | EP300//PRKCQ//KMT2D// |
| GO:0022604 | regulation of cell morphogenesis | Biological process | 9 | 468 | 169 | 17653 | 2.00876194811106 | 0.0364661421279086 | 0.675534243173841 | 1.4381101796855 | 0.0532544378698225 | ARHGAP35//RHOF//CNTN2//SRF//BDNF//SEMA4B//SEMA4G//ZSWIM6//ENAM// |
| GO:0032386 | regulation of intracellular transport | Biological process | 8 | 395 | 169 | 17653 | 2.11555688712456 | 0.0366122528428581 | 0.675534243173841 | 1.43637354707805 | 0.0473372781065089 | IL4R//NUP214//CTDSPL2//CHRM1//NUCB1//CNIH2//PRKAA1//RIMS4// |
| GO:1901564 | organonitrogen compound metabolic process | Biological process | 80 | 7112 | 169 | 17653 | 1.17497886728656 | 0.0367559615931468 | 0.675534243173841 | 1.43467221094636 | 0.473372781065089 | DDAH1//CDKN2D//GRIN2B//NCAM1//SPTBN5//PSMB8//YWHAB//CAMK2A//PRKAA1//UBE4B//MGRN1//COPS7B//ST6GALNAC6//ABHD17B//TULP4//GPC1//NUP214//OGT//NDUFA4//DPYS//CHRM1//STK35//PTPRT//PTPN18//CTDSPL2//LHPP//EP300//FOXO1//HAT1//BTG2//ABCA1//SPRN//PYURF//NUDT16//TRABD2B//ADAM11//SFRP1//CASP7//PRKCQ//TP53INP2//PCTP//PLA2G15//SMPD1//GPD1L//ARHGEF6//ATXN7L3//CRIM1//CNTN2//B4GALT5//ARID4B//HDAC11//USP54//TOB1//PURA//MEX3A//OSR1//RAB3B//ZDHHC22//VAPA//CSPG5//FAF2//FOXP3//MIF//PPP1R26//UBXN2B//ARMC8//TWF1//ENAM//NUCB1//APBB1//SULT4A1//SLC26A1//KMT2D//POLR2E//SH3BP5//NPNT//ISCU//FAM161A//EFNA3//PAX8// |
| GO:0006355 | regulation of transcription, DNA-templated | Biological process | 46 | 3745 | 169 | 17653 | 1.28303299863329 | 0.0368798493569644 | 0.675534243173841 | 1.43321086157645 | 0.272189349112426 | OSR1//EP300//FOXO1//HEYL//ARHGAP35//KCNIP3//APBB1//SP5//FOXP3//BTG2//SRF//BAHD1//KMT2D//HAT1//CARHSP1//HOXC9//ARID4B//ZNF395//PURA//OGT//MICAL2//NDP//ATXN7L3//TP53INP2//GPATCH3//SFRP1//PAX8//YWHAB//BTAF1//TOB1//CTDSPL2//ATP1B4//MCIDAS//LMO2//CREB3L3//PRKCQ//CAMK2A//PSMB8//PRKAA1//NPNT//ZNF618//MYCBP//PFDN1//ASF1B//HDAC11//DYNLL1// |
| GO:0048869 | cellular developmental process | Biological process | 52 | 4323 | 169 | 17653 | 1.25646363814303 | 0.0369009759522899 | 0.675534243173841 | 1.43296214752416 | 0.307692307692308 | CDH7//KMT2D//TP53INP2//SFRP1//SEMA4B//SEMA4G//SRF//OSR1//FOXP3//HEYL//PAX8//DYNLL1//APBB1//EFNA3//GPC1//ARHGAP35//NCAM1//SPTBN5//PRKCQ//BDNF//CNTN2//UNC5A//MIF//RHOF//EP300//ABCA1//TWF1//HDAC11//BTG2//ENAM//CSPG5//FOXO1//KIF2A//MEA1//ASF1B//PURA//CAMK2A//UBE4B//GNAO1//VAPA//KRTAP1-1//FERMT3//PSMB8//IL4R//TOB1//NPNT//P2RX2//NPTX1//ARID4B//LMO2//ZSWIM6//MCIDAS// |
| GO:0007034 | vacuolar transport | Biological process | 4 | 131 | 169 | 17653 | 3.18948461990153 | 0.0372622680405759 | 0.675534243173841 | 1.42873071445509 | 0.0236686390532544 | GOSR2//SPTBN5//VPS53//MGRN1// |
| GO:0001844 | protein insertion into mitochondrial membrane involved in apoptotic signaling pathway | Biological process | 2 | 32 | 169 | 17653 | 6.52847633136095 | 0.0374575397294382 | 0.675534243173841 | 1.42646075117376 | 0.0118343195266272 | YWHAB//DYNLL1// |
| GO:0006335 | DNA replication-dependent nucleosome assembly | Biological process | 2 | 32 | 169 | 17653 | 6.52847633136095 | 0.0374575397294382 | 0.675534243173841 | 1.42646075117376 | 0.0118343195266272 | ASF1B//HAT1// |
| GO:0008038 | neuron recognition | Biological process | 2 | 32 | 169 | 17653 | 6.52847633136095 | 0.0374575397294382 | 0.675534243173841 | 1.42646075117376 | 0.0118343195266272 | ARHGAP35//CNTN2// |
| GO:0034723 | DNA replication-dependent nucleosome organization | Biological process | 2 | 32 | 169 | 17653 | 6.52847633136095 | 0.0374575397294382 | 0.675534243173841 | 1.42646075117376 | 0.0118343195266272 | ASF1B//HAT1// |
| GO:0035587 | purinergic receptor signaling pathway | Biological process | 2 | 32 | 169 | 17653 | 6.52847633136095 | 0.0374575397294382 | 0.675534243173841 | 1.42646075117376 | 0.0118343195266272 | P2RX2//CNTN2// |
| GO:0071526 | semaphorin-plexin signaling pathway | Biological process | 2 | 32 | 169 | 17653 | 6.52847633136095 | 0.0374575397294382 | 0.675534243173841 | 1.42646075117376 | 0.0118343195266272 | SEMA4B//SEMA4G// |
| GO:0061333 | renal tubule morphogenesis | Biological process | 3 | 77 | 169 | 17653 | 4.06969953123799 | 0.0376692899235995 | 0.675749092183829 | 1.42401256628914 | 0.0177514792899408 | NPNT//PAX8//OSR1// |
| GO:0072028 | nephron morphogenesis | Biological process | 3 | 77 | 169 | 17653 | 4.06969953123799 | 0.0376692899235995 | 0.675749092183829 | 1.42401256628914 | 0.0177514792899408 | NPNT//PAX8//OSR1// |
| GO:0018193 | peptidyl-amino acid modification | Biological process | 19 | 1282 | 169 | 17653 | 1.54809423146157 | 0.0380132087674074 | 0.680114631994646 | 1.42006546922455 | 0.112426035502959 | EP300//NUP214//PRKCQ//CAMK2A//OSR1//RAB3B//ZDHHC22//ST6GALNAC6//PRKAA1//GPD1L//MIF//ARID4B//FOXP3//APBB1//HAT1//OGT//SFRP1//KMT2D//SH3BP5// |
| GO:0051050 | positive regulation of transport | Biological process | 15 | 947 | 169 | 17653 | 1.65452409664903 | 0.0384094481371682 | 0.683691701373061 | 1.41556193268654 | 0.0887573964497041 | P2RX2//GPD1L//SCN3B//ABCA1//CYP4F2//DYNLL1//CHRM1//IL4R//CTDSPL2//APBB1//MIF//RAB3B//CAMK2A//YWHAB//PRKAA1// |
| GO:0045934 | negative regulation of nucleobase-containing compound metabolic process | Biological process | 21 | 1457 | 169 | 17653 | 1.50553743811756 | 0.0386346630844444 | 0.683691701373061 | 1.41302287068968 | 0.124260355029586 | OSR1//EP300//FOXO1//HEYL//ARHGAP35//KCNIP3//APBB1//SP5//FOXP3//BTG2//BAHD1//KMT2D//HAT1//PURA//SFRP1//YWHAB//BTAF1//TOB1//SRF//NUDT16//SHQ1// |
| GO:0014033 | neural crest cell differentiation | Biological process | 3 | 78 | 169 | 17653 | 4.01752389622212 | 0.03890911809953 | 0.683691701373061 | 1.40994861265162 | 0.0177514792899408 | SEMA4B//SEMA4G//SFRP1// |
| GO:1900034 | regulation of cellular response to heat | Biological process | 3 | 78 | 169 | 17653 | 4.01752389622212 | 0.03890911809953 | 0.683691701373061 | 1.40994861265162 | 0.0177514792899408 | EP300//NUP214//CAMK2A// |
| GO:0001101 | response to acid chemical | Biological process | 7 | 329 | 169 | 17653 | 2.22246002769734 | 0.0392927900659158 | 0.683691701373061 | 1.40568713211437 | 0.0414201183431953 | SH3BP4//CDKN2D//OSR1//ABCA1//PRKAA1//SFRP1//FOXO1// |
| GO:0031328 | positive regulation of cellular biosynthetic process | Biological process | 25 | 1812 | 169 | 17653 | 1.44116475305981 | 0.0393133704418455 | 0.683691701373061 | 1.40545972134025 | 0.14792899408284 | SRF//EP300//HEYL//MICAL2//FOXO1//APBB1//NDP//FOXP3//ATXN7L3//TP53INP2//GPATCH3//SFRP1//PAX8//PRKCQ//DDAH1//PRKAA1//OSR1//ATP1B4//MCIDAS//LMO2//ARID4B//KMT2D//CREB3L3//OGT//NPNT// |
| GO:0071705 | nitrogen compound transport | Biological process | 30 | 2264 | 169 | 17653 | 1.38412925753236 | 0.0395846719480774 | 0.683691701373061 | 1.40247294985854 | 0.177514792899408 | SPRN//NUP214//SRSF3//YWHAB//ZDHHC22//GOSR2//TOMM40//ARFIP2//RABL3//KCNIP3//RAB3B//MLPH//CPLX1//VPS37D//VPS53//FAF2//DYNLL1//SFRP1//CTDSPL2//ABCA1//FOXP3//APBB1//MIF//IL4R//CHRM1//RALBP1//NUCB1//PRKAA1//OSR1//PAX8// |
| GO:0007274 | neuromuscular synaptic transmission | Biological process | 2 | 33 | 169 | 17653 | 6.3306437152591 | 0.0396284410673133 | 0.683691701373061 | 1.40199301192471 | 0.0118343195266272 | CHRM1//P2RX2// |
| GO:0018345 | protein palmitoylation | Biological process | 2 | 33 | 169 | 17653 | 6.3306437152591 | 0.0396284410673133 | 0.683691701373061 | 1.40199301192471 | 0.0118343195266272 | ZDHHC22//ABHD17B// |
| GO:0032633 | interleukin-4 production | Biological process | 2 | 33 | 169 | 17653 | 6.3306437152591 | 0.0396284410673133 | 0.683691701373061 | 1.40199301192471 | 0.0118343195266272 | FOXP3//PRKCQ// |
| GO:0033144 | negative regulation of intracellular steroid hormone receptor signaling pathway | Biological process | 2 | 33 | 169 | 17653 | 6.3306437152591 | 0.0396284410673133 | 0.683691701373061 | 1.40199301192471 | 0.0118343195266272 | HEYL//SFRP1// |
| GO:0043114 | regulation of vascular permeability | Biological process | 2 | 33 | 169 | 17653 | 6.3306437152591 | 0.0396284410673133 | 0.683691701373061 | 1.40199301192471 | 0.0118343195266272 | DDAH1//ARHGAP35// |
| GO:0048846 | axon extension involved in axon guidance | Biological process | 2 | 33 | 169 | 17653 | 6.3306437152591 | 0.0396284410673133 | 0.683691701373061 | 1.40199301192471 | 0.0118343195266272 | SEMA4B//SEMA4G// |
| GO:1902284 | neuron projection extension involved in neuron projection guidance | Biological process | 2 | 33 | 169 | 17653 | 6.3306437152591 | 0.0396284410673133 | 0.683691701373061 | 1.40199301192471 | 0.0118343195266272 | SEMA4B//SEMA4G// |
| GO:1903506 | regulation of nucleic acid-templated transcription | Biological process | 46 | 3766 | 169 | 17653 | 1.2758785395331 | 0.0401531917304767 | 0.689509386797332 | 1.39627992745029 | 0.272189349112426 | OSR1//EP300//FOXO1//HEYL//ARHGAP35//KCNIP3//APBB1//SP5//FOXP3//BTG2//SRF//BAHD1//KMT2D//HAT1//ZNF618//ATP1B4//MYCBP//PFDN1//ASF1B//PRKCQ//ZNF395//HDAC11//DYNLL1//CARHSP1//HOXC9//ARID4B//PURA//OGT//MICAL2//NDP//ATXN7L3//TP53INP2//GPATCH3//SFRP1//PAX8//YWHAB//BTAF1//TOB1//CTDSPL2//MCIDAS//LMO2//CREB3L3//CAMK2A//PSMB8//PRKAA1//NPNT// |
| GO:0046902 | regulation of mitochondrial membrane permeability | Biological process | 3 | 79 | 169 | 17653 | 3.96666916335855 | 0.0401695546943884 | 0.689509386797332 | 1.39610298267813 | 0.0177514792899408 | YWHAB//DYNLL1//CAMK2A// |
| GO:0032956 | regulation of actin cytoskeleton organization | Biological process | 7 | 331 | 169 | 17653 | 2.20903126620068 | 0.0403803606173607 | 0.691373111025849 | 1.39382980690227 | 0.0414201183431953 | TWF1//ARHGAP35//BRK1//ARFIP2//SFRP1//SPTBN5//RHOF// |
| GO:0065008 | regulation of biological quality | Biological process | 46 | 3770 | 169 | 17653 | 1.27452482224978 | 0.0408015794808599 | 0.695342158284053 | 1.38932302449482 | 0.272189349112426 | FOXO1//FOXP3//TP53INP2//GPD1L//MIF//P2RX2//CHRM1//DDAH1//CYP4F2//TWF1//AKAP10//ISCU//ATP1B4//CPLX1//ARHGAP35//BRK1//RHOF//SCN3B//SRF//ABCA1//EP300//DPYS//PRKCQ//CNTN2//ARFIP2//DYNLL1//SLC26A1//RIMS4//CNIH2//PRKAA1//CARHSP1//PSMB8//YWHAB//NUP214//SFRP1//CAMK2A//GRIN2B//BDNF//SEMA4B//SEMA4G//RAB3B//SPTBN5//ANO3//FERMT3//ABHD17B//PAX8// |
| GO:0009411 | response to UV | Biological process | 4 | 135 | 169 | 17653 | 3.09498137190445 | 0.0408875715735756 | 0.695342158284053 | 1.38840868263506 | 0.0236686390532544 | EP300//UBE4B//CDKN2D//PRKAA1// |
| GO:0032880 | regulation of protein localization | Biological process | 15 | 956 | 169 | 17653 | 1.63894803297764 | 0.0411643824735668 | 0.695342158284053 | 1.38547839526118 | 0.0887573964497041 | DYNLL1//SFRP1//CTDSPL2//FOXP3//APBB1//MIF//IL4R//CHRM1//YWHAB//ABHD17B//NUCB1//TP53INP2//PRKAA1//BDNF//GPD1L// |
| GO:0048194 | Golgi vesicle budding | Biological process | 3 | 80 | 169 | 17653 | 3.91708579881657 | 0.0414505148637208 | 0.695342158284053 | 1.38247007063641 | 0.0177514792899408 | CNIH2//GOSR2//VAPA// |
| GO:2001141 | regulation of RNA biosynthetic process | Biological process | 46 | 3774 | 169 | 17653 | 1.27317397453144 | 0.0414580806628638 | 0.695342158284053 | 1.3823908078095 | 0.272189349112426 | OSR1//EP300//FOXO1//HEYL//ARHGAP35//KCNIP3//APBB1//SP5//FOXP3//BTG2//SRF//BAHD1//KMT2D//HAT1//ZNF618//ATP1B4//MYCBP//PFDN1//ASF1B//PRKCQ//ZNF395//HDAC11//DYNLL1//CARHSP1//HOXC9//ARID4B//PURA//OGT//MICAL2//NDP//ATXN7L3//TP53INP2//GPATCH3//SFRP1//PAX8//YWHAB//BTAF1//TOB1//CTDSPL2//MCIDAS//LMO2//CREB3L3//CAMK2A//PSMB8//PRKAA1//NPNT// |
| GO:0003002 | regionalization | Biological process | 7 | 333 | 169 | 17653 | 2.19576381114843 | 0.0414874563293145 | 0.695342158284053 | 1.38208319173182 | 0.0414201183431953 | EP300//SFRP1//HOXC9//BTG2//PAX8//OSR1//SRF// |
| GO:0018130 | heterocycle biosynthetic process | Biological process | 54 | 4550 | 169 | 17653 | 1.23969308797711 | 0.0417107674209105 | 0.695342158284053 | 1.37975181965787 | 0.319526627218935 | OSR1//EP300//FOXO1//HEYL//ARHGAP35//KCNIP3//APBB1//SP5//FOXP3//BTG2//CDKN2D//SRF//POLR2E//PAX8//CREB3L3//POLR3H//SRSF3//NUP214//OGT//BAHD1//KMT2D//HAT1//ZNF618//ATP1B4//MYCBP//HOXC9//MCIDAS//ARID4B//PRKAA1//ASF1B//ZNF395//ATXN7L3//PURA//TP53INP2//GPATCH3//HDAC11//DYNLL1//PFDN1//PRKCQ//CARHSP1//LHPP//MICAL2//NDP//SFRP1//YWHAB//BTAF1//TOB1//CTDSPL2//NUDT16//LMO2//SLC26A1//CAMK2A//PSMB8//NPNT// |
| GO:0010591 | regulation of lamellipodium assembly | Biological process | 2 | 34 | 169 | 17653 | 6.14444831186913 | 0.0418459645751308 | 0.695342158284053 | 1.37834641693606 | 0.0118343195266272 | BRK1//TWF1// |
| GO:0045987 | positive regulation of smooth muscle contraction | Biological process | 2 | 34 | 169 | 17653 | 6.14444831186913 | 0.0418459645751308 | 0.695342158284053 | 1.37834641693606 | 0.0118343195266272 | NPNT//SRF// |
| GO:0061384 | heart trabecula morphogenesis | Biological process | 2 | 34 | 169 | 17653 | 6.14444831186913 | 0.0418459645751308 | 0.695342158284053 | 1.37834641693606 | 0.0118343195266272 | UBE4B//SRF// |
| GO:1900077 | negative regulation of cellular response to insulin stimulus | Biological process | 2 | 34 | 169 | 17653 | 6.14444831186913 | 0.0418459645751308 | 0.695342158284053 | 1.37834641693606 | 0.0118343195266272 | PRKCQ//PRKAA1// |
| GO:2000008 | regulation of protein localization to cell surface | Biological process | 2 | 34 | 169 | 17653 | 6.14444831186913 | 0.0418459645751308 | 0.695342158284053 | 1.37834641693606 | 0.0118343195266272 | GPD1L//BDNF// |
| GO:0042157 | lipoprotein metabolic process | Biological process | 5 | 198 | 169 | 17653 | 2.63776821469129 | 0.0419517036151143 | 0.695390616541711 | 1.37725039822931 | 0.029585798816568 | ABHD17B//ABCA1//SPRN//PYURF//ZDHHC22// |
| GO:0010629 | negative regulation of gene expression | Biological process | 27 | 2005 | 169 | 17653 | 1.40663430181942 | 0.0423346072401212 | 0.696984513111005 | 1.37330446504644 | 0.159763313609467 | OSR1//EP300//FOXO1//HEYL//ARHGAP35//KCNIP3//APBB1//SP5//FOXP3//BTG2//BAHD1//KMT2D//HAT1//NUDT16//PURA//SFRP1//YWHAB//BTAF1//TOB1//MEX3A//CARHSP1//PSMB8//NUP214//POLR2E//SRF//SHQ1//MIF// |
| GO:0006914 | autophagy | Biological process | 9 | 482 | 169 | 17653 | 1.95041616538584 | 0.0426422633431106 | 0.696984513111005 | 1.37015975212087 | 0.0532544378698225 | UBXN2B//TP53INP2//EP300//FOXO1//SH3BP4//PRKAA1//TOMM40//VPS37D//DYNLL1// |
| GO:0061919 | process utilizing autophagic mechanism | Biological process | 9 | 482 | 169 | 17653 | 1.95041616538584 | 0.0426422633431106 | 0.696984513111005 | 1.37015975212087 | 0.0532544378698225 | UBXN2B//TP53INP2//FOXO1//EP300//SH3BP4//PRKAA1//TOMM40//VPS37D//DYNLL1// |
| GO:0007188 | adenylate cyclase-modulating G-protein coupled receptor signaling pathway | Biological process | 5 | 199 | 169 | 17653 | 2.62451309803455 | 0.042713614437791 | 0.696984513111005 | 1.36943367689979 | 0.029585798816568 | ABCA1//GPR3//GNAZ//CHRM1//GNAO1// |
| GO:0035303 | regulation of dephosphorylation | Biological process | 5 | 199 | 169 | 17653 | 2.62451309803455 | 0.042713614437791 | 0.696984513111005 | 1.36943367689979 | 0.029585798816568 | NPNT//PPP1R26//SMPD1//YWHAB//UBXN2B// |
| GO:0071456 | cellular response to hypoxia | Biological process | 5 | 199 | 169 | 17653 | 2.62451309803455 | 0.042713614437791 | 0.696984513111005 | 1.36943367689979 | 0.029585798816568 | EP300//PSMB8//DDAH1//PRKAA1//SFRP1// |
| GO:0072006 | nephron development | Biological process | 4 | 137 | 169 | 17653 | 3.04979916209562 | 0.042772844971706 | 0.696984513111005 | 1.36883186233562 | 0.0236686390532544 | NPNT//PAX8//HEYL//OSR1// |
| GO:0019438 | aromatic compound biosynthetic process | Biological process | 54 | 4558 | 169 | 17653 | 1.23751723350063 | 0.042961805219232 | 0.696984513111005 | 1.3669174782331 | 0.319526627218935 | OSR1//EP300//FOXO1//HEYL//ARHGAP35//KCNIP3//APBB1//SP5//FOXP3//BTG2//CDKN2D//SRF//POLR2E//PAX8//CREB3L3//POLR3H//SRSF3//NUP214//OGT//BAHD1//KMT2D//HAT1//ZNF618//ATP1B4//MYCBP//HOXC9//MCIDAS//ARID4B//PRKAA1//ASF1B//ZNF395//ATXN7L3//PURA//TP53INP2//GPATCH3//HDAC11//DYNLL1//PFDN1//PRKCQ//CARHSP1//LHPP//MICAL2//NDP//SFRP1//YWHAB//BTAF1//TOB1//CTDSPL2//NUDT16//LMO2//SLC26A1//CAMK2A//PSMB8//NPNT// |
| GO:0071229 | cellular response to acid chemical | Biological process | 5 | 200 | 169 | 17653 | 2.61139053254438 | 0.0434838483380921 | 0.696984513111005 | 1.3616720276483 | 0.029585798816568 | SH3BP4//OSR1//ABCA1//PRKAA1//SFRP1// |
| GO:0045944 | positive regulation of transcription by RNA polymerase II | Biological process | 17 | 1132 | 169 | 17653 | 1.56867982520334 | 0.0436696659315918 | 0.696984513111005 | 1.35982013037363 | 0.100591715976331 | SRF//EP300//HEYL//MICAL2//CREB3L3//NPNT//OSR1//FOXO1//ATP1B4//APBB1//MCIDAS//LMO2//FOXP3//ARID4B//PAX8//KMT2D//OGT// |
| GO:0030518 | intracellular steroid hormone receptor signaling pathway | Biological process | 4 | 138 | 169 | 17653 | 3.0276991681674 | 0.0437336367361808 | 0.696984513111005 | 1.35918440674423 | 0.0236686390532544 | KMT2D//EP300//HEYL//SFRP1// |
| GO:0072332 | intrinsic apoptotic signaling pathway by p53 class mediator | Biological process | 3 | 82 | 169 | 17653 | 3.82154712079665 | 0.0440736456257043 | 0.696984513111005 | 1.35582102460596 | 0.0177514792899408 | EP300//MIF//ZNF385B// |
| GO:0002823 | negative regulation of adaptive immune response based on somatic recombination of immune receptors built from immunoglobulin superfamily domains | Biological process | 2 | 35 | 169 | 17653 | 5.96889264581572 | 0.0441090302545483 | 0.696984513111005 | 1.35547249016374 | 0.0118343195266272 | FOXP3//IL4R// |
| GO:0010742 | macrophage derived foam cell differentiation | Biological process | 2 | 35 | 169 | 17653 | 5.96889264581572 | 0.0441090302545483 | 0.696984513111005 | 1.35547249016374 | 0.0118343195266272 | ABCA1//EP300// |
| GO:0030517 | negative regulation of axon extension | Biological process | 2 | 35 | 169 | 17653 | 5.96889264581572 | 0.0441090302545483 | 0.696984513111005 | 1.35547249016374 | 0.0118343195266272 | SEMA4B//SEMA4G// |
| GO:0044331 | cell-cell adhesion mediated by cadherin | Biological process | 2 | 35 | 169 | 17653 | 5.96889264581572 | 0.0441090302545483 | 0.696984513111005 | 1.35547249016374 | 0.0118343195266272 | DENND6A//CDH7// |
| GO:0051204 | protein insertion into mitochondrial membrane | Biological process | 2 | 35 | 169 | 17653 | 5.96889264581572 | 0.0441090302545483 | 0.696984513111005 | 1.35547249016374 | 0.0118343195266272 | YWHAB//DYNLL1// |
| GO:0086005 | ventricular cardiac muscle cell action potential | Biological process | 2 | 35 | 169 | 17653 | 5.96889264581572 | 0.0441090302545483 | 0.696984513111005 | 1.35547249016374 | 0.0118343195266272 | GPD1L//SCN3B// |
| GO:0090077 | foam cell differentiation | Biological process | 2 | 35 | 169 | 17653 | 5.96889264581572 | 0.0441090302545483 | 0.696984513111005 | 1.35547249016374 | 0.0118343195266272 | EP300//ABCA1// |
| GO:0098751 | bone cell development | Biological process | 2 | 35 | 169 | 17653 | 5.96889264581572 | 0.0441090302545483 | 0.696984513111005 | 1.35547249016374 | 0.0118343195266272 | EP300//SRF// |
| GO:0001819 | positive regulation of cytokine production | Biological process | 8 | 411 | 169 | 17653 | 2.03319944139708 | 0.0444859528762093 | 0.700134646551103 | 1.35177710253423 | 0.0473372781065089 | POLR3H//EP300//POLR2E//PRKCQ//FOXP3//MIF//GPATCH3//IL4R// |
| GO:0097435 | supramolecular fiber organization | Biological process | 11 | 641 | 169 | 17653 | 1.79253016274497 | 0.044515436643054 | 0.700134646551103 | 1.35148936235885 | 0.0650887573964497 | RHOF//TWF1//MICAL2//BRK1//CGNL1//KIF2A//ARHGAP35//ARFIP2//SRF//SFRP1//SPTBN5// |
| GO:0051056 | regulation of small GTPase mediated signal transduction | Biological process | 7 | 339 | 169 | 17653 | 2.15690073484491 | 0.0449268561496269 | 0.704965958561315 | 1.34749397100501 | 0.0414201183431953 | ABCA1//OGT//ARHGEF6//ARHGAP35//CGNL1//RALBP1//RHOF// |
| GO:0044070 | regulation of anion transport | Biological process | 3 | 83 | 169 | 17653 | 3.77550438440151 | 0.0454156259762088 | 0.710985829808102 | 1.34279469543267 | 0.0177514792899408 | CYP4F2//MIF//OSR1// |
| GO:0048762 | mesenchymal cell differentiation | Biological process | 5 | 203 | 169 | 17653 | 2.57279855423091 | 0.0458445909463459 | 0.713443845363272 | 1.33871189795975 | 0.029585798816568 | SEMA4B//SEMA4G//HEYL//SFRP1//OSR1// |
| GO:0009891 | positive regulation of biosynthetic process | Biological process | 25 | 1841 | 169 | 17653 | 1.41846308122997 | 0.0460744502953527 | 0.713443845363272 | 1.33653983755477 | 0.14792899408284 | SRF//EP300//HEYL//MICAL2//FOXO1//APBB1//NDP//FOXP3//ATXN7L3//TP53INP2//GPATCH3//SFRP1//PAX8//PRKCQ//DDAH1//PRKAA1//OSR1//ATP1B4//MCIDAS//LMO2//ARID4B//KMT2D//CREB3L3//OGT//NPNT// |
| GO:0003179 | heart valve morphogenesis | Biological process | 2 | 36 | 169 | 17653 | 5.80309007232084 | 0.0464165742954073 | 0.713443845363272 | 1.33332691514036 | 0.0118343195266272 | HEYL//FGFRL1// |
| GO:0033280 | response to vitamin D | Biological process | 2 | 36 | 169 | 17653 | 5.80309007232084 | 0.0464165742954073 | 0.713443845363272 | 1.33332691514036 | 0.0118343195266272 | SFRP1//CDKN2D// |
| GO:0034314 | Arp2/3 complex-mediated actin nucleation | Biological process | 2 | 36 | 169 | 17653 | 5.80309007232084 | 0.0464165742954073 | 0.713443845363272 | 1.33332691514036 | 0.0118343195266272 | ARFIP2//BRK1// |
| GO:0035418 | protein localization to synapse | Biological process | 2 | 36 | 169 | 17653 | 5.80309007232084 | 0.0464165742954073 | 0.713443845363272 | 1.33332691514036 | 0.0118343195266272 | ABHD17B//BSN// |
| GO:0050919 | negative chemotaxis | Biological process | 2 | 36 | 169 | 17653 | 5.80309007232084 | 0.0464165742954073 | 0.713443845363272 | 1.33332691514036 | 0.0118343195266272 | SEMA4B//SEMA4G// |
| GO:0051693 | actin filament capping | Biological process | 2 | 36 | 169 | 17653 | 5.80309007232084 | 0.0464165742954073 | 0.713443845363272 | 1.33332691514036 | 0.0118343195266272 | TWF1//SPTBN5// |
| GO:0019932 | second-messenger-mediated signaling | Biological process | 7 | 342 | 169 | 17653 | 2.13798055296031 | 0.046713548387679 | 0.716380335024655 | 1.33055714220359 | 0.0414201183431953 | ABCA1//GPR3//GRIN2B//DDAH1//MGRN1//P2RX2//PRKAA1// |
| GO:0090287 | regulation of cellular response to growth factor stimulus | Biological process | 6 | 272 | 169 | 17653 | 2.30416811695092 | 0.0471182636505993 | 0.720952074816749 | 1.32681072205919 | 0.0355029585798817 | NPNT//FAM89B//TOB1//CTDSPL2//SFRP1//GPC1// |
| GO:0006888 | ER to Golgi vesicle-mediated transport | Biological process | 5 | 205 | 169 | 17653 | 2.5476980805311 | 0.047460208561227 | 0.724544899547581 | 1.32367035763172 | 0.029585798816568 | CNIH2//GOSR2//VAPA//SPTBN5//DYNLL1// |
| GO:0002028 | regulation of sodium ion transport | Biological process | 3 | 85 | 169 | 17653 | 3.68666898712148 | 0.0481599119944966 | 0.729679055294171 | 1.31731431536193 | 0.0177514792899408 | GPD1L//SCN3B//OSR1// |
| GO:0016575 | histone deacetylation | Biological process | 3 | 85 | 169 | 17653 | 3.68666898712148 | 0.0481599119944966 | 0.729679055294171 | 1.31731431536193 | 0.0177514792899408 | FOXP3//HDAC11//ARID4B// |
| GO:0030516 | regulation of axon extension | Biological process | 3 | 85 | 169 | 17653 | 3.68666898712148 | 0.0481599119944966 | 0.729679055294171 | 1.31731431536193 | 0.0177514792899408 | SRF//SEMA4B//SEMA4G// |
| GO:0036294 | cellular response to decreased oxygen levels | Biological process | 5 | 206 | 169 | 17653 | 2.53533061412076 | 0.0482805764889407 | 0.729679055294171 | 1.31622755289799 | 0.029585798816568 | EP300//PSMB8//PRKAA1//SFRP1//DDAH1// |
| GO:0009132 | nucleoside diphosphate metabolic process | Biological process | 4 | 143 | 169 | 17653 | 2.92183556088882 | 0.0487189373180557 | 0.729679055294171 | 1.31230219332136 | 0.0236686390532544 | NUP214//OGT//PRKAA1//NUDT16// |
| GO:0009395 | phospholipid catabolic process | Biological process | 2 | 37 | 169 | 17653 | 5.64624980009595 | 0.048767548867805 | 0.729679055294171 | 1.31186907217014 | 0.0118343195266272 | SMPD1//PLA2G15// |
| GO:0043370 | regulation of CD4-positive, alpha-beta T cell differentiation | Biological process | 2 | 37 | 169 | 17653 | 5.64624980009595 | 0.048767548867805 | 0.729679055294171 | 1.31186907217014 | 0.0118343195266272 | FOXP3//IL4R// |
| GO:0045581 | negative regulation of T cell differentiation | Biological process | 2 | 37 | 169 | 17653 | 5.64624980009595 | 0.048767548867805 | 0.729679055294171 | 1.31186907217014 | 0.0118343195266272 | IL4R//FOXP3// |
| GO:1902229 | regulation of intrinsic apoptotic signaling pathway in response to DNA damage | Biological process | 2 | 37 | 169 | 17653 | 5.64624980009595 | 0.048767548867805 | 0.729679055294171 | 1.31186907217014 | 0.0118343195266272 | MIF//CDKN2D// |
| GO:0050767 | regulation of neurogenesis | Biological process | 12 | 732 | 169 | 17653 | 1.71238723445533 | 0.0490574743364091 | 0.731503837044705 | 1.30929481467011 | 0.0710059171597633 | SFRP1//ARHGAP35//APBB1//TWF1//CNTN2//EFNA3//BDNF//HEYL//SRF//SEMA4B//SEMA4G//ZSWIM6// |
| GO:0001666 | response to hypoxia | Biological process | 7 | 346 | 169 | 17653 | 2.1132640147758 | 0.0491658982880861 | 0.731503837044705 | 1.30833602162959 | 0.0414201183431953 | EP300//PSMB8//PRKAA1//SFRP1//DDAH1//P2RX2//SRF// |
| GO:1901698 | response to nitrogen compound | Biological process | 16 | 1065 | 169 | 17653 | 1.56928632941634 | 0.0494090521474706 | 0.731503837044705 | 1.30619347744172 | 0.0946745562130177 | CHRM1//FOXO1//SH3BP4//UBE4B//FAF2//PRKAA1//OGT//P2RX2//CAMK2A//SMPD1//GNAO1//BTG2//PRKCQ//ABCA1//SFRP1//CASP7// |
| GO:1901655 | cellular response to ketone | Biological process | 3 | 86 | 169 | 17653 | 3.64380074308518 | 0.0495620046985347 | 0.731503837044705 | 1.30485113546983 | 0.0177514792899408 | PRKAA1//SFRP1//FOXO1// |
| GO:0051128 | regulation of cellular component organization | Biological process | 31 | 2403 | 169 | 17653 | 1.34753402428424 | 0.0497413427869705 | 0.731503837044705 | 1.30328249495872 | 0.183431952662722 | CRIM1//PRKCQ//TWF1//ARHGAP35//BRK1//RHOF//SFRP1//APBB1//ENAM//CDKN2D//SH3BP4//FOXP3//CNTN2//EP300//TRABD2B//ARFIP2//GPR3//SRF//BDNF//SEMA4B//SEMA4G//SPTBN5//OGT//ABCA1//YWHAB//DYNLL1//IL4R//ZSWIM6//ABHD17B//GRIN2B//PRKAA1// |
| GO:0035148 | tube formation | Biological process | 4 | 144 | 169 | 17653 | 2.90154503616042 | 0.0497522030093885 | 0.731503837044705 | 1.30318768409135 | 0.0236686390532544 | ARHGAP35//OSR1//PAX8//SFRP1// |
| GO:0043271 | negative regulation of ion transport | Biological process | 4 | 144 | 169 | 17653 | 2.90154503616042 | 0.0497522030093885 | 0.731503837044705 | 1.30318768409135 | 0.0236686390532544 | CYP4F2//GNAO1//SESTD1//OSR1// |
| GO:1903829 | positive regulation of cellular protein localization | Biological process | 7 | 347 | 169 | 17653 | 2.10717391675051 | 0.0497915645169381 | 0.731503837044705 | 1.30284422740202 | 0.0414201183431953 | CTDSPL2//CHRM1//YWHAB//DYNLL1//PRKAA1//ABHD17B//GPD1L// |
